# A Structure-guided Active Site Affinity Ligand Unmasks a Stress-Sensitizing Role for BLVRB in the Endoplasmic Reticulum

**DOI:** 10.1021/acs.jmedchem.6c01466

**Published:** 2026-07-27

**Authors:** Rahul Raghavan Thekke Veedu, Jawaad Sheriff, Natasha M. Nesbitt, Natalia Marchenko, Lisa Pennacchia, Tiziana Ginex, Patrick Hearing, Dale F. Kreitler, Wadie F. Bahou

**Affiliations:** † Blood Cell Technologies, JLABS@NYC, 101 Sixth Avenue, New York, New York 10013, United States; ‡ Department of Medicine, 12301 Stony Brook University, Stony Brook, New York 11794, United States; § Deparment of Pathology, Stony Brook University, Stony Brook, New York 11794, United States; ∥ Pharmacelera, Parc Científic de Barcelona (PCB), Baldiri Reixac 4-8, 08028 Barcelona, Spain; ⊥ Deparment of Microbiology and Immunology, Stony Brook University, Stony Brook, New York 11794, United States; # Center for Biomolecular Structure, Brookhaven National Laboratory, Upton, New York 11793, United States

## Abstract

Biliverdin IXb reductase (BLVRB) is an NAD­(P)­H-dependent oxidoreductase that regulates hematopoiesis and cellular stress, although measurement and sequelae of cellular active site engagement remain undefined. Here, we report the development of a nanoBRET platform enabling real-time BLVRB target engagement. Structure-guided design and chemical syntheses of BODIPY-labeled pyrazolopyrimidinone inhibitors generate cell-permeable acceptor ligands retaining high-affinity binding to the BLVRB active site. *In vitro* and cellular nanoBRET assays demonstrate specific energy transfer and inform equilibrium binding affinities, target engagement, and residence time analyses for diverse panels of BLVRB inhibitors. NanoBRET demonstrates strong concordance with enzymatic inhibition and is validated by crystallographic structures confirming active site binding. Live-cell imaging using affinity ligands reveals predominant endoplasmic reticulum localization and transient suppression of the ER stress chaperone GRP78/BiP without eliciting a canonical unfolded protein response. These studies inform a redox-regulated mechanism whereby spatiotemporal BLVRB active site engagement functions as a stress sensitizer modulating ER proteostasis.

## Graphical abstract

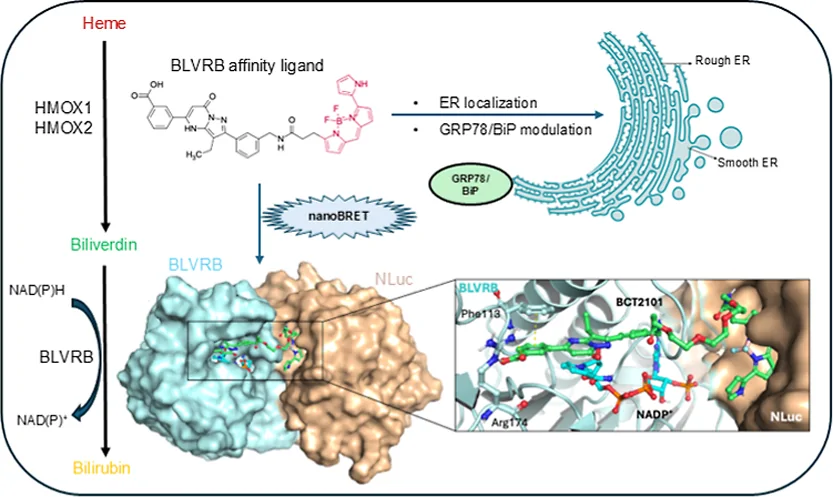

## Introduction

The heme prosthetic group (Fe^[2+]^-protoporphyrin IX) functions in redox homeostasis for a broad spectrum of phylogenetically enriched hemoproteins that maintain gas transport/exchange, catalysis, and the electron transport chain (ETC).^1,2^ Cellular heme is toxic and its balance is coordinated by synthetic and degradation pathways that provide energy and cytoprotection from metabolic stress.^3^ Heme biosynthesis is generated from tricarboxylic acid-derived carbon fuels (glucose and glutamine), and is ultimately coupled to the heme degradation pathway [heme → biliverdin (BV) → bilirubin (BR)] for sequential catalysis by heme oxygenases (HMOX1, HMOX2) and two nonoverlapping biliverdin reductases (BLVRA, BLVRB).^4,5^ Both BLVRA and BLVRB function in NADP­(H)-dependent catabolic processes coupled to cellular antioxidant functions.^3,6^ BLVRA retains specificity for the predominant BV IXα in adults,^7^ while BLVRB is promiscuous and catalyzes the reduction of BVs (IXβ, IXγ, IXδ),^7–10^ flavins,^11^ and ferric ion.^12^ Bilirubin is a lipophilic tetrapyrrole that retains potent antioxidant and cytoprotective functions,^5,6^ extending beyond the glutathione (GSSG/GSH) couple.^6^ A BLVRB ancillary function as an *S*-nitroso-CoA-dependent nitrosyl transferase has also been proposed.^13^

Targeted genetic disruption of *Blvra* and *Blvrb* in mice establish that neither gene is required for organ development,^2,14^ and both protein products display dispensable function(s) in physiological homeostasis. Erythrocytes abundantly express BLVRB as a nonphysiological methemoglobin reductase,^11^ although it functions as the target redox coupler in cytochrome b5 reductase-deficient (*CYB5R3*) patients with congenital methemoglobinemia (Hb^[Fe3+]^). In contrast, BLVRB has been implicated in stress-regulated hematopoietic pathways that differentially regulate thrombopoietic responses,^15,16^ and hematopoietic lineage fate.^17,18^ Furthermore, BLVRB-selective small molecule inhibitors promote chemotherapy stress-induced megakaryocyte expansion *in vitro* and *in vivo* when delivered into mice,^19^ highlighting a BLVRB function regulated by cellular stress.

In this manuscript, we report the development of high-quality chemical probes for BLVRB that enable quantitative measurement of target engagement across biochemical and cellular systems. Guided by BLVRB active-site structural features, we derivatized a pyrazolopyrimidinone inhibitor scaffold to generate fluorescent, cell-permeable ligands that retain potent and selective binding to the catalytic pocket. These ligands function as efficient nanoBRET acceptors when paired with BLVRB–nanoLuciferase (nanoLuc) fusion proteins, and inform a robust and concentration-dependent biophysical readout of active-site engagement. Assay validations is demonstrated in a reconstituted (recombinant) system and translated to live-cell formats using ectopically expressed BLVRB–nanoLuc chimers. Competitive displacement studies employing a chemically diverse panel of BLVRB inhibitors demonstrated strong concordance between nanoBRET-derived binding affinities and enzymatic inhibition, while revealing compound-dependent differences in target residence times and megakaryocyte-promoting effects using hematopoietic stem cell models. Extending beyond target engagement quantification, the fluorescent probe enabled direct visualization of BLVRB engagement in living cells, revealing preferential localization to the endoplasmic reticulum (ER). Consistent with this spatially restricted engagement, selective BLVRB inhibition modulated the ER stress chaperone GRP78/BiP without eliciting a canonical unfolded protein response. Together, these studies define a robust chemical probe–based nanoBRET platform for interrogating BLVRB structure–activity relationships and support a redox-regulated model in which spatiotemporally constrained BLVRB activity sensitizes ER proteostasis pathways.

## Results

### Design and Chemical Syntheses of BLVRB nanoBRET Ligand Acceptors

Customized BRET donor/acceptor pairs for direct BLVRB target engagement required generation of (1) BLVRB/NLuc (nanoluciferase) chimers (as donor), and (2) a fluorescently tagged small molecule retaining capacity to bind BLVRB (acceptor). We previously applied computational and medicinal chemistry to *de novo* synthesize and characterize novel small molecules targeting the BLVRB active site. Structure/activity relationships (SARS) of >100 small molecules established that two compound classes (diazabicyclic or pyrazolopyrimidinone derivatives) retaining drug-like characteristics inhibited BLVRB redox activity and bound the BLVRB active site as established by NMR and crystallographic studies.^19^ We used the previously identified small molecule BLVRB inhibitor (BCT2029) as scaffold for the ligand acceptor. This pyrazolopyrimidinone derivative inhibits BLVRB reductive activity using both FMN (flavin mononucleotide; IC_50_ 194 ± 6.5 M^–9^) and DCPIP (2,6-dichlorophenolindophenol; IC_50_ 28 ± 6 M^–9^) as substrates.^20^ Structural data confirmed that the ternary BLVRB/NADP^+^/BCT2029 crystal (PDB 9C16) structure unambiguously localized to the BLVRB active site adjacent to the NADP nicotinamide ring.^19^

We applied the BODIPY^TMR^ (tetramethylrhodamine) red-shifted fluorophore as a nanoluciferase acceptor,^21^ and synthesized two distinct BCT2029_BODIPY derivatives, differentiated by the presence (BCT2101: BCT2029_PEG_BODIPY) or absence (BCT2102: BCT2029_BODIPY) of a polyethylene glycol (PEG) linker.^22^ Synthesis of the pyrazolo-pyrimidine BODIPY derivatives is schematized in Figure 1A, B (details are provided in **Experimental Methods**), and commenced from commercially available 3-cyanobenzoic acid (**1**), which was converted in a four-step one-pot sequence: (*i*) Fischer esterification (EtOH, H_2_SO_4_, reflux), (*ii*) Claisen-type condensation with butyronitrile (nPrCN, nBuLi, THF, −78 °C), (*iii*) cyclocondensation with hydrazine (NH_2_NH_2_, MeOH, 100 °C), and (*iv*) nitrile reduction (LiAlH_4_, THF, rt) to afford the diaminopyrazole benzylamine intermediate (**2**). Condensation of (**2**) with ethyl (*m*-methoxycarbonylbenzoyl)­acetate (**3**) in acetic acid at 140 °C furnished the fully elaborated pyrazolo-pyrimidine core (**4**), bearing both a methyl ester and a free primary amine available for downstream conjugation. For the synthesis of BCT2101, compound (**4**) was coupled to Boc-protected PEG_4_ linker (**5**) via HATU/DIPEA-mediated amide bond formation (DMF, rt) to give intermediate (**6**). Sequential saponification of the methyl ester (LiOH, MeOH/THF/H_2_O, rt) and Boc deprotection (HCl, dioxane/DCM, rt) afforded the bifunctional intermediate (**8**), exposing a free carboxylic acid and a terminal amine. Conjugation of **(8)** with the BODIPY succinimidyl ester **(9)** (5-[3-(2,5-dioxo-1-pyrrolidinyloxy)-3-oxopropyl]-4,4-difluoro-3-(2-pyrrolyl)-4H-3a,4a-diaza-4-bora-*s*-indacen-3a-ium-4-uide) using DIPEA in DMF followed by preparative HPLC purification and lyophilization, gave BCT2101 as a blue solid with an overall yield of 12.6%. The shorter analog BCT2102 was prepared in a single step by direct conjugation of parent inhibitor scaffold **(4)** with BODIPY succinimidyl ester **(9)** (DIPEA, DMF, dark, rt), affording **BCT2102** as a blue solid after preparative HPLC purification (overall yield of 14.8%). Structures were confirmed by ^1^H NMR and LC/MS/MS, and were determined to be >95% pure by HPLC analysis (Supporting file Figures S1–S6). Both compounds displayed robust fluorescent emission peaks of 605 nm (Figure 1C, D), and in HEK293 cells ectopically expressing the BLVRB_NLuc chimer (HEK293/BLVRB_NLuc, *vide infra*), both acceptor ligands (BCT2101 and BCT2102) exhibited dose-dependent, rapid membrane permeability and cellular residence within 1 h as established by flow cytometry (Figure 1E).

**Figure 1:**
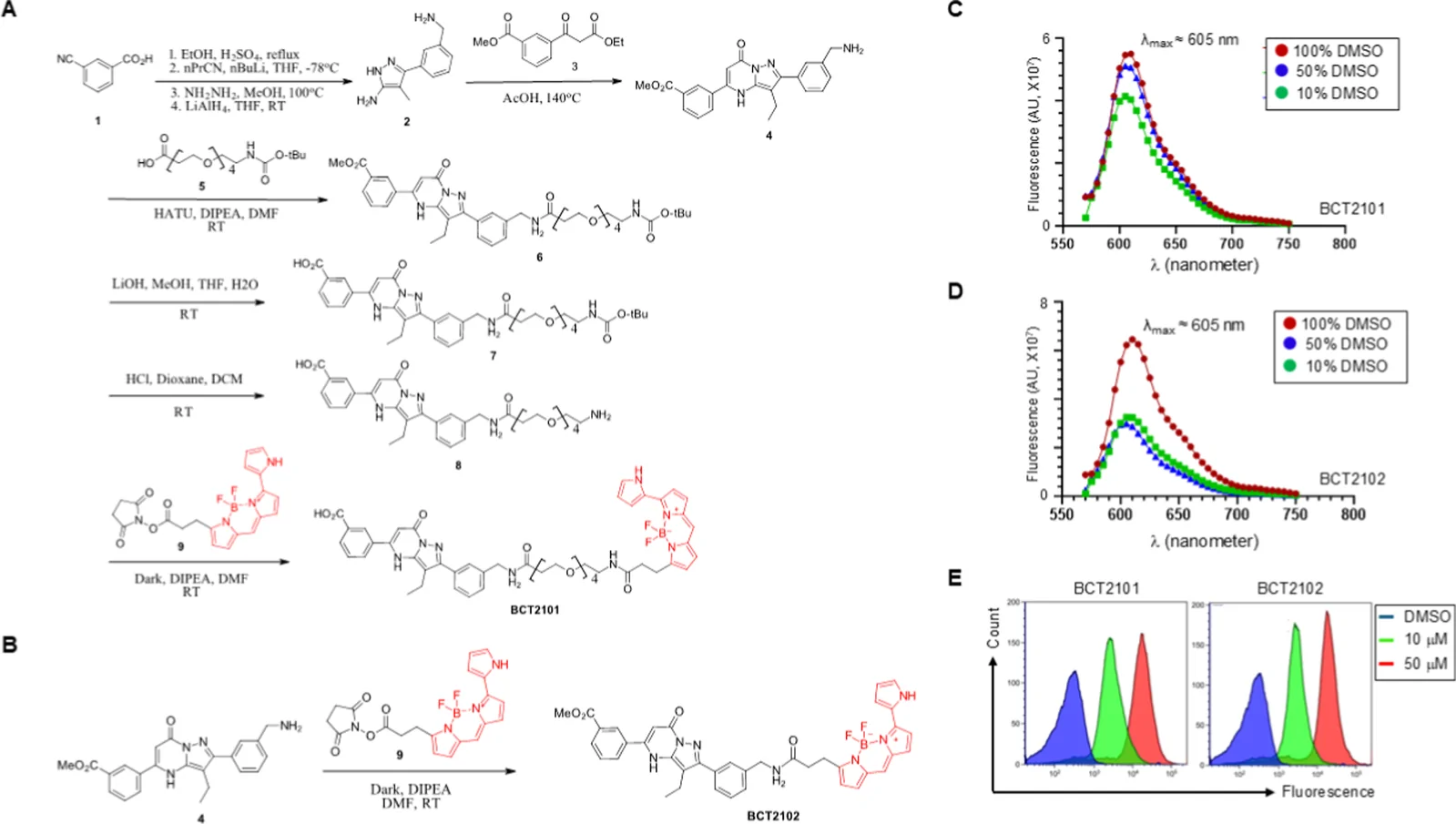
. Design and chemical structures of BCT2029-derived BODIPY probes for nanoBRET-based BLVRB target engagement. **A, B.** Schema shows the key chemical intermediates and steps required for generation of BCT2101 (BCT2029_PEG_BODIPY, *Panel A*) or BCT2102 (BCT2029_BODIPY, *Panel B*); in both *panels*, the BODIPY fluorophore is highlighted in red. **C, D**. BCT2101 (*Panel A*) or BCT2102 (*Panel B*) fluorescent spectra are displayed in various dilutions of DMSO, demonstrating the identical λ_max_ peak ∼605 nm. **E.** HEK293 cells ectopically expressing BLVRB_NLuc (HEK293/BLVRB_NLuc cells, 1 × 10^5^/mL) were incubated for 1 h at 37 °C with dose-modified BCT2101 or BCT2102 (or *v/v*-equivalent DMSO as control), followed by detachment and flow cytometric detection using a 561 nm excitation laser and 585/42 nm bandpass filter capable of light detection at 605 nm. Results are from a single experiment, repeated twice.

### Structure-guided Modeling of BLVRB Active Site Engagement Using nanoBRET

We applied computational modeling to support the nanoBRET design and predict potentially favorable donor–acceptor 3-dimensional geometries for BLVRB NanoBRET functional engagement. Computational modeling was completed using both N- (BLVRB_NLuc) and C- (NLuc_BLVRB) terminal chimers, and both ligand acceptors (Figure 2A). *In silico* docking established that the selected fluorophore attachment site for both acceptors could preserve proximity to the NLuc’s catalytic residue (Gln^12^) and the fluorophore for both N- and C-terminal BLVRB chimers (Figure 2B,C). Specifically, the BODIPY (4,4-difluoro-4-bora-3a,4a-diaza-*s*-indacene) moiety of BCT2101/BCT2102 was located 1.00/1.10 nm and 1.80/1.10 nm from Gln^12^ in the NLuc_BLVRB and BLVRB_NLuc chimeras, respectively (Figure S7). The BLVRB-selective ligand incorporated in both acceptor constructs is stably anchored within the BLVRB active site. Within the benzoic acid moiety, the carboxylate forms a salt-bridge interaction with Arg^174^, while the benzene ring participates in a π–π stacking interaction with Phe^113^ (Figure 2C). The BODIPY group extends outward from the BLVRB binding pocket and packs against the surface of the NLuc protein. Binding modes of both chimers is consistent with that reported for the BCT2029 scaffold as previously assessed using BLVRB docking and MD (molecular dynamics) simulations.^19^ Both ligands are predicted to be within the 10 nm active range for nanoBRET,^22^ with no critical computationally restricted preference for N- or C-terminal chimers.

**Figure 2:**
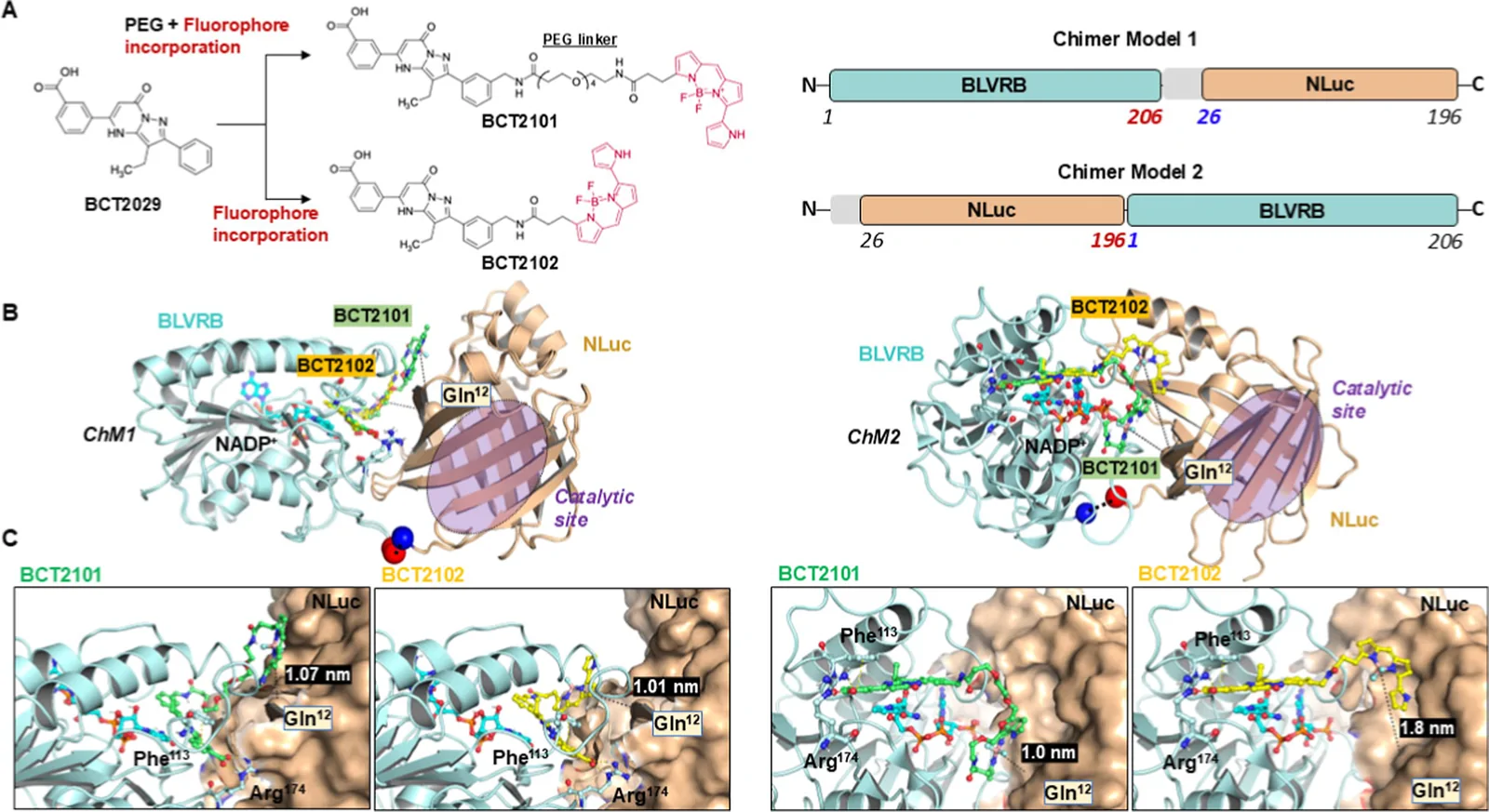
Structure-guided modeling supports favorable donor–acceptor geometry for BLVRB nanoBRET assays. **A.** Schema demonstrates key components if designer/acceptor pairs encompassing affinity ligands and BLVRB/nanoluciferase (NLuc) chimers BLVRB_NLuc (chimer model 1, ChM1) or NLuc_BLVRB (chimer model 2, ChM2) applied for modeling and subsequent validation. Affinity ligands BCT2101 or BCT2102 targeting the BLVRB active site are modeled on the selective BLVRB small molecule BCT2029, differentiated by presence or absence of a polyethylene glycol (PEG) linker. Amino acids residues comprising BLVRB (residues 1–206) or the NLuc catalytic subunit (residues 26–196) are shown (complete sequences are provided in Supplementary Table S1). **B, C**. Models (ribbon, *Panel B*) and expanded (*Panel C*) were generated using BLVRB (UniProt ID P30043, PDB 5OOH) and nanoluciferase (UniProt ID Q9GV45, PDB ID 5IBO); key BLVRB active site residues Arg^174^ and Phe^113^ are shown, and docked poses of acceptor ligands BCT2101, BCT2102, and NADP^+^ are represented as green, yellow, and blue sticks, respectively; the nanoluciferase catalytic site is marked with a violet circle (*Panel B*). The N- and C-terminal residues (Cα carbon) applied to generate the fused chimeric systems are highlighted as blue and red spheres, respectively (*Panel B*). Position of the catalytic NLuc residue, Gln^12^ is highlighted with a wheat sphere (*Panel B*). Distances between Gln^12^ (Cα carbon atom) and the fluorophore moiety (B atom) of BCT2101 and BCT2102 are highlighted with black dashed lines (*Panel C*).

Comparison of the *apo* BLVRB structure (PDB ID 8ELL, resolution 1.52 Å), the cofactor-bound BLVRB structure (PDB ID 1HDO, resolution 1.15 Å), and the ternary BLVRB/NADP^+^/BCT2029 crystal structure (PDB ID 9C16, resolution 1.58 Å) revealed a stable and well-defined protein architecture. Relative to the *apo* structure, the cofactor-bound and ternary complexes exhibited global RMSD values of 0.25 Å and 0.34 Å (respectively), indicating only minor structural deviations upon ligand binding. These results suggest that the overall BLVRB fold remains largely conserved across the different structural states (Figures S8A, B). Nevertheless, localized conformational changes were observed in the solvent-exposed loop regions 120 (residues 116–124) and 170 (residues 168–75) upon cofactor binding. A more detailed analysis of the binding site revealed distinct side-chain arrangements for Trp^116^ (loop 120), Arg^170^, and Arg^174^ (loop 170) when comparing the cofactor-bound BLVRB structure with the ternary BLVRB/NADP^+^/BCT2029 complex, imputing conformational adjustments necessary to accommodate BCT2029. Arg^174^ adopts a binding-competent orientation that enables the formation of a salt bridge with the acidic group of BCT2029. In addition, Arg^174^ engages in an edge-to-face π-stacking interaction with Phe^113^, which in turn forms a π–π stacking interaction with the aromatic ring of the benzoic acid moiety of BCT2029. Together, these interactions are expected to contribute to the stabilization of the inhibitor within the binding site and highlight the structural rearrangements required for productive ligand recognition (Figure S8C).

### Conserved Active Site Binding of Structurally Diverse Inhibitors

Previous crystallographic studies established active site binding for BCT2029 and the diazabicyclic derivative (BCT1028),^19,23^ and we obtained crystallographic data to further define structural binding features for a subset of newly characterized pyrazolopyrimidinone inhibitors (BCT2066, BCT2104, BCT2109) studied by nanoBRET (*vide infra*). Ternary crystal structures of BLVRB/NADP^+^/BCT2066 (PDB 13EA), BLVRB/NADP^+^/BCT2104 (PDB 13DZ), and BLVRB/NADP^+^/BCT2109 (PDB 13EB) were determined at 1.7 Å, 1.7 Å, and 1.8 Å, respectively (Table S1 provides crystallographic refinement characteristics). Overall structures were nearly identical to ligand-free BLVRB (PDB entry 1HDO, 204 aligned C-alpha atoms),^24^ and electron densities were clear in the molecular replacement maps. All ligands localized unambiguously to the BLVRB active site; ring stacking between the nicotinamide moiety of the NADP^+^ cofactor and small molecules was evident, comparable to FMN.^24^ In each structure, the *meta* benzoic acid phenyl ring forms salt-bridging interactions with Arg^174^ (similar to that previously identified for BCT2029),^19^ and the His^153^ imidazole forms an H-bonding interaction with the pyrimidinone carbonyl (Figure 3 A–C). Overlays with the parent BCT2029 scaffold (PDB entry 9C16, Figure 3 D–F) demonstrate that each ligand adopts a nearly identical pose to that of BCT2029 for the *m*-benzoic acid and pyrimidinone ring portion of the scaffold. The substituted-phenyl (BCT2104, BCT2109) or furan (BCT2066) rings adjacent to the nicotinamide ribosyl ring of NADP are rotated slightly out of the plane relative to the phenyl ring of BCT2029; for BCT2104, additional H-bonding opportunities are presented by the disubstituted urea and *meta*-methoxy groups that could form transient interactions with the side chains of neighboring Asn^79^ and Gln^14^ residues (Figures 3E and S9). These data provide structural confirmation for direct BLVRB active site binding and target engagement specificity using BCT2029_BODIPY acceptor ligands.

**Figure 3:**
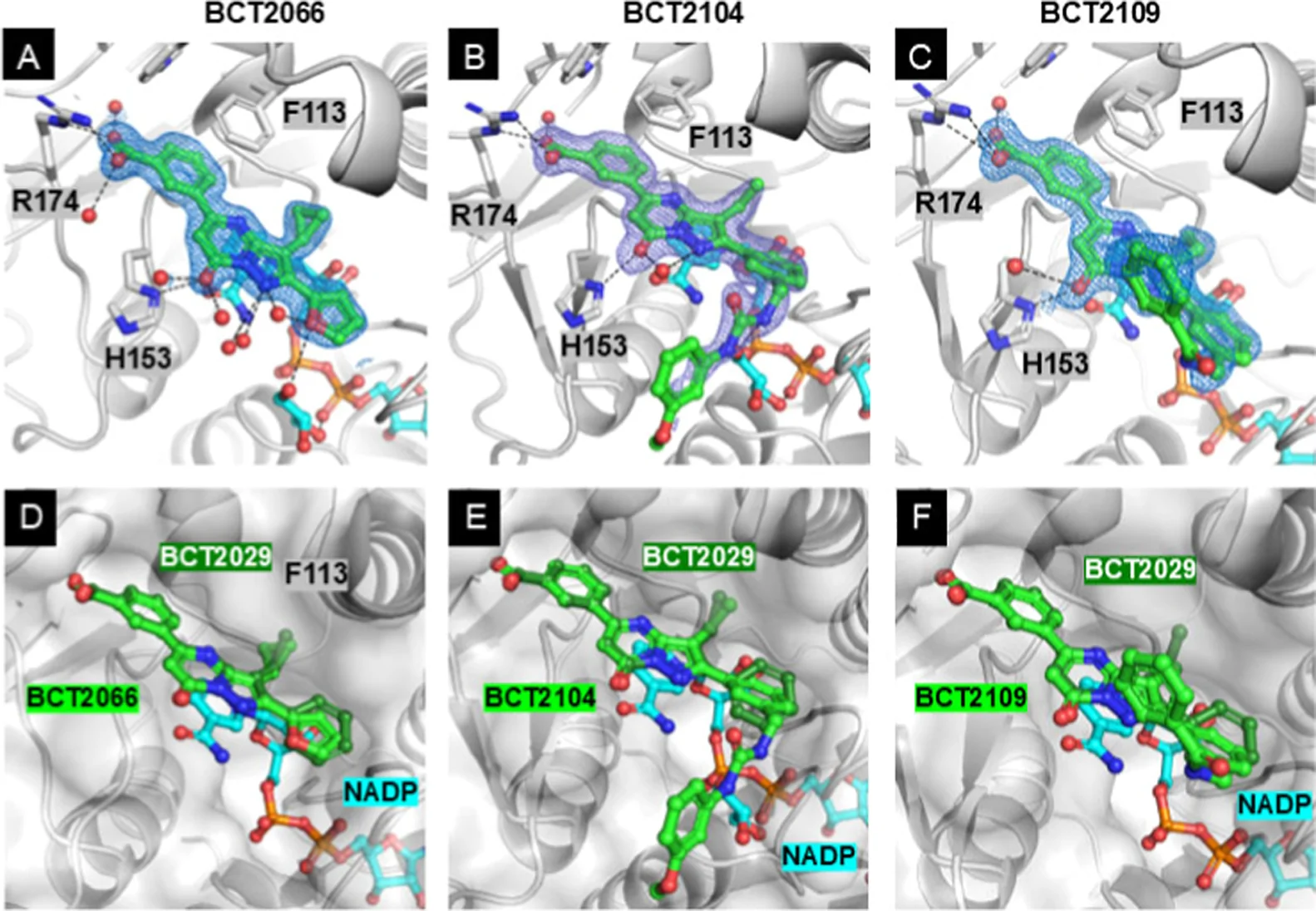
Crystal structures of BLVRB/NADP^+^ ternary complexes confirm conserved active site binding. **A**–**C**. Ternary cocrystal structures of BLVRB/NADP^+^/BCT2066 (PDB 13EA), BLVRB/NADP^+^/BCT2104 (PDB 13DZ), and BLVRB/NADP^+^/BCT2109 (PDB 13EB) were determined at 1.7 Å, 1.7 Å, and 1.8 Å respectively, and localize unambiguously to the BLVRB active site.^24^ Shown are the electron density (blue mesh) contours at 1.2 σ generated from 2*m*Fo-*D*Fc weighted map coefficients. Ordered solvent molecules are drawn as red spheres; H-bonding and salt-bridging interactions are represented as dashed lines. In each structure, the *meta* benzoic acid phenyl ring forms salt-bridging interactions with R174, and the H153 imidazole forms an H-bonding interaction with the pyrimidinone carbonyl.^19^ BCT2066 and BCT2104 structures feature a glycerol molecule (cyan) adjacent to the active site which forms an H-bonding interaction with the adenosine ribosyl 5′-phopshate. Ring stacking between the nicotinamide moiety of the NADP^+^ cofactor and small molecules is evident.^24^ **D**–**F.** Overlays of the parent BCT2029 (dark green) scaffold (PDB entry 9C16) with BCT2066 (*Panel D*, light green), BCT2104 (*Panel E*, light green), and BCT2109 (*Panel E*, light green) demonstrate that each ligand adopts a nearly identical pose to that of BCT2029 for the *m*-benzoic acid and pyrimidinone ring portion of the scaffold. The substituted-phenyl (BCT2104, BCT2109) or furan (BCT2066) rings adjacent to the nicotinamide ribosyl ring of NADP (cyan) are rotated slightly out of the plane relative to the phenyl ring of BCT2029 (cyan). Overlays were generated by aligning C-α reference positions (S111, F113, T129, H132, P152, R174) with 9C16. BCT2104 displays additional H-bonding opportunities presented by the disubstituted urea and *meta*-methoxy groups, features that could form transient interactions with the side chains of neighboring N79 and Q14 residues (see Figure S9).

### BLVRB/NLuc Chimers Retain Enzymatic Activity Comparable to Native BLVRB

We compared the functional characteristics of BLVRB_NLuc and NLuc_BLVRB using codon-optimized recombinant chimers expressed and purified as His_6_-tagged proteins in *E. coli*. Chimers were configured with either N- or C-terminal BLVRB (amino acids 1–206)^24^ fused with the *Oplophorus gracilirostris* nanoluciferase catalytic subunit (amino acids 26–196).^25^ No amino acid changes were introduced within either protein backbone (chimer sequence characteristics provided in Tables S2 and S3), and Coomassie-stained gels confirmed size-fidelity of the predicted BLVRB-NLuc and NLuc-BLVRB chimers (∼40 kDa) (Figure 4 A,B). Both chimers retained equimolar-comparable luminescence using nanoluciferace-specific furimazine as substrate, with no detectable bioluminescence using BLVRB alone (Figure 4C). Similarly, BLVRB reductase activity using DCPIP (DR activity, Figure 4D) or FMN (FR activity, Figure 4E) as substrates^20^ were comparable between the two chimers, and for DCPIP were nearly identical to pure BLVRB (Figure 4D). Statistically lower flavin reductase activity was evident for NLuc_BLVRB compared to BLVRB (*p* < 0.05), although FR activity of BLVRB_NLuc was not statistically different from that of BLVRB (Figure 4E). DCPIP inhibitory IC_50_s using both acceptor ligands (BCT2101 and BCT2102) were nearly identical between BLVRB_NLluc and native BLVRB (Figure 4F,G), confirming a functionally competent active site unaffected by the nanoluciferase moiety. Similarly, the BRET signal using BLVRB_NLuc confirmed energy transfer using both BRET acceptors (Figure S10), and we applied the probe titration data (reported in milliBRET (mBRET) units^22^) to estimate equilibrium dissociation constants (*K*_*d*_) of 0.6 ± 0.1 μM for BCT2101 and 0.8 ± 0.2 μM for BCT2102) Binding specificity using a saturating concentration of unlabeled BCT2029 (5 μM) confirmed dose-dependent attenuation of the BRET signal for both ligands (Figures 4H,I; S10).

**Figure 4:**
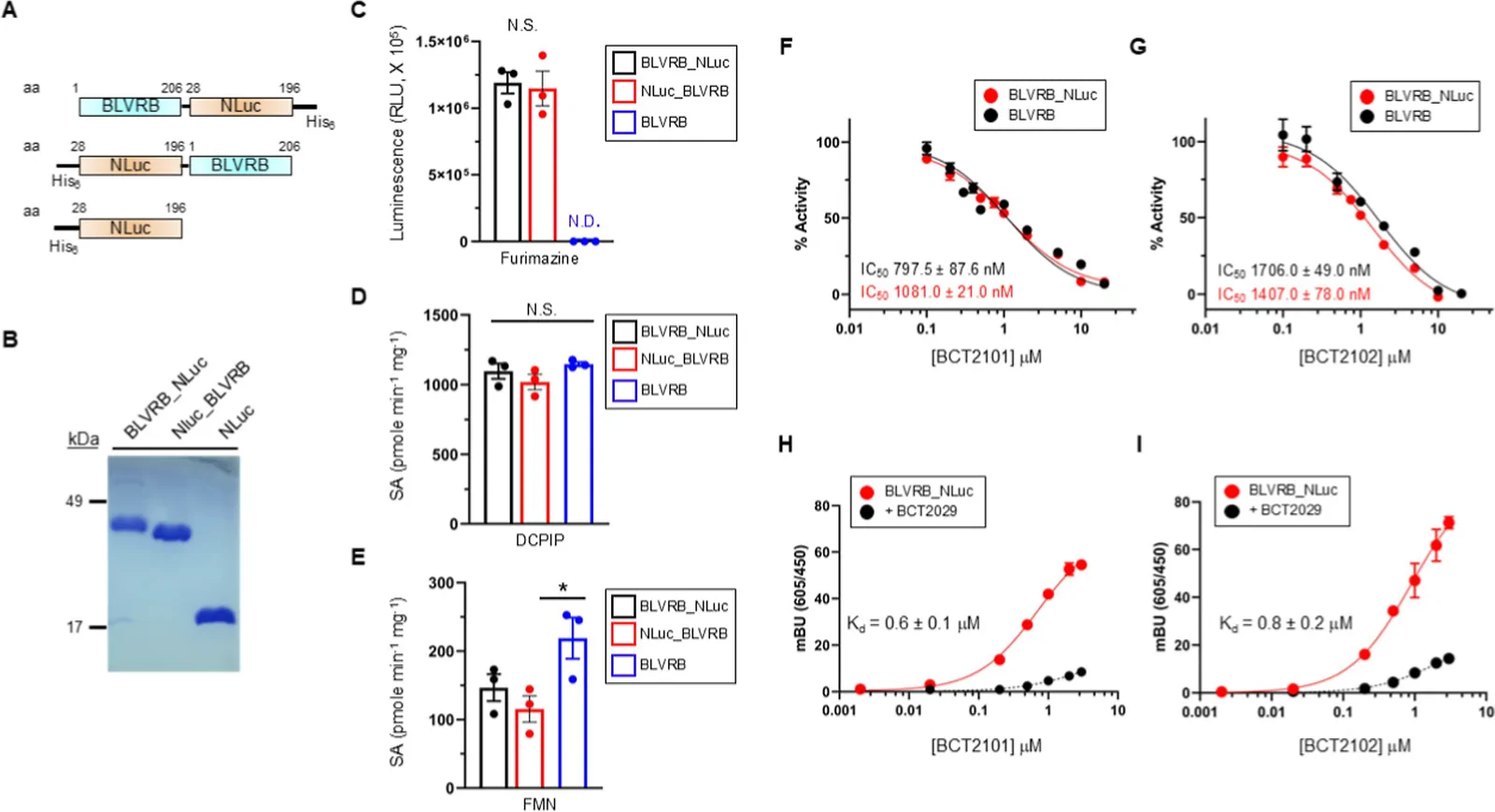
BLVRB_nanoLuc chimers retain enzymatic activity and small molecule inhibition comparable to native BLVRB. **A, B.** Schemas (*Panel A*) and purification (Coomassie-stained gel, 20 ng/lane; *Panel B*) of recombinant proteins (BLVRB_NLuc, NLuc_BLVRB, NLuc) used for studies are shown. **C**–**E.** Recombinant proteins (BLVRB_NLuc, NLuc_BLVRB, BLVRB [50 nM]) were used for determination of nanoluciferase activity (*Panel C*), or active site reduction of BLVRB substrates DCPIP (DR activity, *Panel D*) or FMN (FR activity, *Panel E*). All data luminescence, specific activity [SA] are the mean ± SEM from *N* = 3 independent replicates; p-value (* <0.05) using unpaired *t*-test; N.S. not significant. **F, G.** Recombinant BLVRB or BLVRB_NLuc (50 nM) were incubated with dose-dependent BCT2101 (*Panel F*) or BCT2102 (*Panel G*), followed by DCPIP reduction (DR) activity assays; IC_50_ determinations were generated from *N* = 3 independent replicates. **H, I.** Recombinant BLVRB_NLuc (50 nM) was incubated with dose-dependent BCT2101 (*Panel H*) or BCT2102 (*Panel I*) for 30 min at 25 °C in the absence or presence of 5 μM unlabeled BCT2029, followed by quantification of donor/acceptor target engagement expressed in milliBRET units (mBU). Apparent tracer affinity values were determined from saturation binding by fitting specific binding values (total minus nonspecific) to a one-site saturation model (data are the mean ± SEM from *N* = 3 independent experiments).

### nanoBRET Provides a Quantitative Measure of BLVRB Active Site Engagement

Functional nanoBRET as a quantifiable parameter of BLVRB active site binding was established using both purified recombinant (rBRET) and cell-based (cBRET) competition target engagement (TE) assays. Cellular validation studies included both nonhematopoietic (HEK293) and hematopoietic (human erythroleukemia, HEL) models,^26,27^ genetically modified to ectopically express BLVRB_NLuc (designated HEK293/BLVRB_NLuc and HEL/BLVRB_NLuc). Cellular BRET in HEK293/BLVRB_NLuc cells established apparent K_d_ of 2.2 ± 1.0 μM (for BCT2101) and K_d_ of 2.5 ± 0.8 μM (for BCT2102, Figure S11). These results were less robust than those in HEL/BLVRB_NLuc cells, which yielded acceptor/donor binding affinities (K_d_) of 0.69 ± 12 μM (for BCT2101), and K_d_ = 0.43 ± 0.08 μM) (for BCT2102, Figure 5A); indeed, results in HEL/BLVRB_NLuc cells were nearly identical to those established for rBRET (see Figure 4, *vide supra*). Ectopic BLVRB_NLuc expression in HEL was ∼2-fold greater than that of endogenous BLVRB as established by immunoblot in vector control (HEL_C) cells (Figure 5B). As expected, statistically greater immunofluorescence intensity was evident in HEL/BLVRB_NLuc cells (compared to HEL_C control cells) using BCT2101 as affinity ligand (*p* < 0.0001, Figure 5C), further establishing target selectivity for ligand binding.

**Figure 5:**
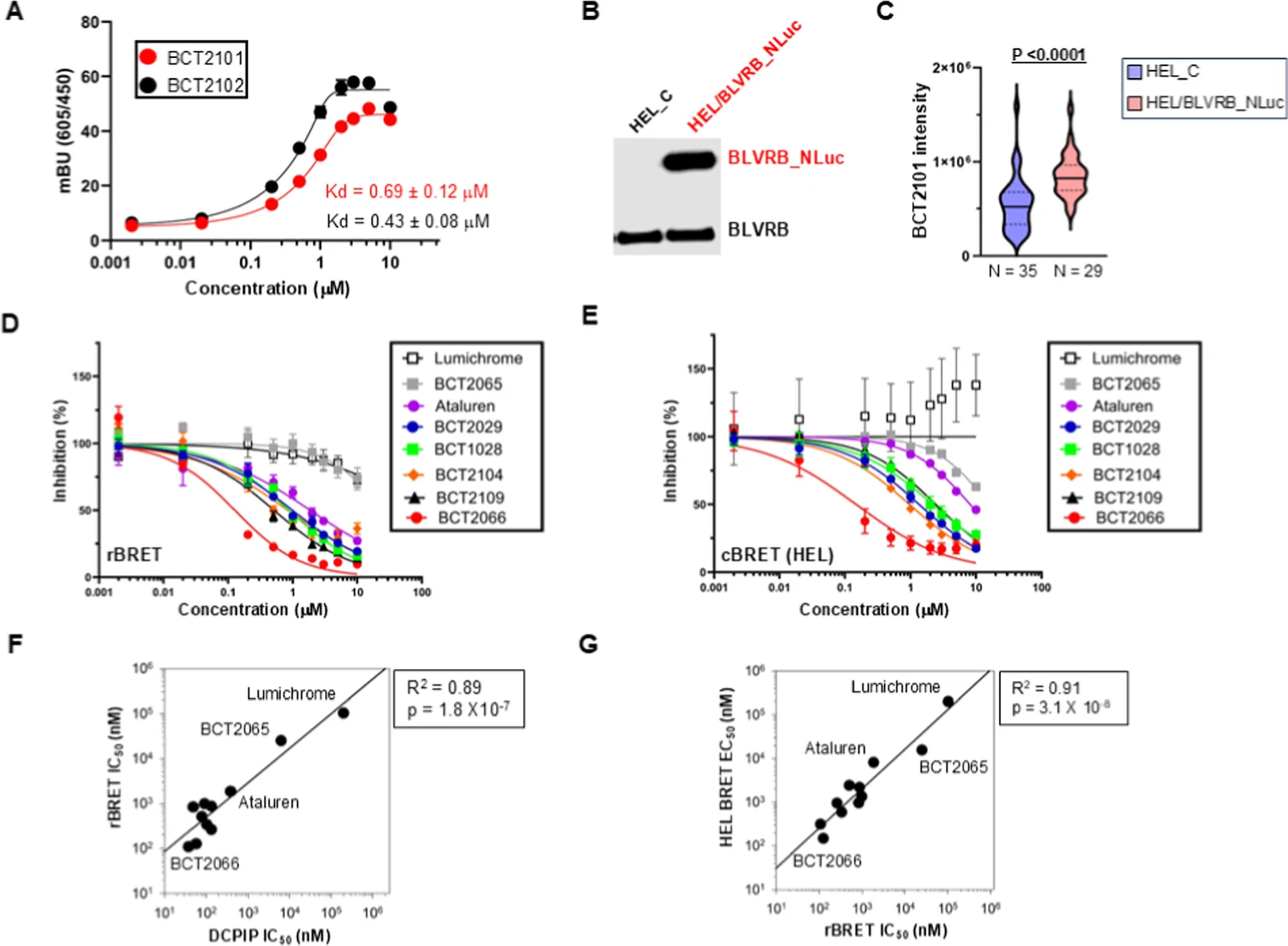
Recombinant and cellular nanoBRET quantifies BLVRB active site engagement and correlates with biochemical inhibition. **A.** HEL/BLVRB_NLuc cells (5 × 10^4^ cells/well) were incubated with dose-modified BCT 2101 or BCT2102 for 2 h at 37 °C, followed by nanoBRET determination of apparent K_d_ (expressed as mean ± SEM from *N* = 3 independent experiments). **B.** Immunoblot (20 μg/lane) was completed using HEL control (HEL_C), or HEL/BLVRB_NLuc cells, probed for BLVRB expression. **C.** Cells (HEL_C, HEL/BLVRB_NLuc, 1 × 10^5^/mL) were incubated with BCT2101 (2 μM) for 60 min at 37 °C, washed, and image capture completed by confocal microscopy for intensity quantification using ImageJ software. Violin plots display the median, interquartile range, and intensity distributions from two distinct experiments (*N* = number of cells analyzed); p-value determined using unpaired *t*-test. **D, E** Competition target engagement assays were completed using biochemically pure recombinant BRET (rBRET [50 nM BLVRB_NLuc], *Panel D*), or HEL/BLVRB_NLuc cells cBRET (5 × 10^4^ cells/well, *Panel E*); Shown are a subset of *N* = 8 (of 11) small molecule inhibitors, presented as normalized dose-dependent inhibition (mean ± SEM) from *N* = 3 independent experiments. **F, G.** Between-assay association plots (rBRET, DCPIP, cBRET) for *N* = 11 small molecule inhibitors confirm highly significant correlations (Pearson) across functional (DCPIP), recombinant (rBRET) and cellular (HEL/BLVRB_NLuc) platforms; p-values are shown.

Competition target engagement (TE) using a subset of previously characterized BLVRB small molecule inhibitors was applied for assay validation, comparing DCPIP, recombinant (rBRET), and cell-based (cBRET; HEL/BLVRB_NLuc) determinations. Compound characteristics are provided in Table S4, and include active site inhibitors^19^ encompassing five pyrazolopyrimidinones (BCT2065, BCT2066, BCT2029, BCT2104, BCT2109), four diazabicyclic analogs displaying preference for Arg^174^ (BCT1028, BCT1061, BCT1070, BCT1072), a high-affinity reference molecule (ataluren),^23^ or the weak/inactive inhibitor lumichrome.^24^ Across the 11-member small molecule subset (Figure 5D–G), between-assay correlations demonstrated highly significant rBRET:DCPIP concordance (R^2^ = 0.89, p-value = 1.8 × 10^–7^, Figure 5F) and rBRET:cBRET concordance (R^2^ = 0.91, p-value = 3.1 × 10^–8^, Figure 5G). Across the assays (DCPIP, rBRET, cBRET), BCT2066 consistently demonstrated potent inhibition, structurally consistent with the overlapping binding characteristics (with BCT2029) established by crystallization (Figure 3, *vide supra*); expectedly, limited (if any) inhibition was evident using lumichrome. These results collectively established consistent and discriminatory binding specificity within the BLVRB active site using either biochemically pure rBRET or *in vitro* cellular cBRET assays.

### Drug residence Time and *in Vitro* Megakaryocytopoiesis

We determined the on-target residence time (RT) to better define pharmacological target engagement as an ancillary parameter predicting biological efficacy, distinct from the IC_50_ which measures binding at equilibrium.^28^ Quantification was completed in HEL/BLVRB_NLuc cells preincubated with individual compounds at their respective IC_50_s, followed by affinity displacement using saturating BCT2101. Relative RT determinations across the N = 11 small molecule inhibitors demonstrated modest (∼3.5 fold) differences for the majority of compounds (range ∼48.0 ± 11.6 min [Ataluren] to ∼168.5 ± 20.1 min [BCT2065]); in contrast, we observed strikingly prolonged RT extension using BCT2104 (∼625.0 ± 143.9 min, Figure 6A and Table S4). To better dissect relative contribution(s) of inhibitor profiles and drug residence times (RT) as parameters of megakaryocyte (MKs) differentiation *in vitro*,^19^ we applied a CD34^+^ hematopoietic stem cell (HSC) model and a fixed dose (10 μM) of inhibitors exceeding the DCPIP IC_50_ for all compounds. We compared megakaryocyte-promoting effects in (CD34^+^) HSCs differentiated in thrombopoietin (TPO)-directed suspension cultures, and quantified relative expansion of CD41a^+^ (integrin α_IIB_) MKs across the inhibitor subset (Figure 6B,C); in the absence of TPO, MKs fail to differentiate.^19^ Statistically significant MK expansion was evident using BCT1028 (p-value <0.0001), BCT1072 (p-value <0.0001), and BCT2065 (*p* <0.001), with no MK-promoting effects using BCT1061, BCT2066, Ataluren, and BCT2029; results with BCT1028, BCT2066 and ataluren are identical to those previously reported.^19^ In contrast, statistically significant CD41a^+^ MK contraction was evident using the long-resident time inhibitor BCT2104 (p-value <0.0001), and also seen using BCT1070 (p-value <0.0001), and BCT2109 (p-value <0.01). While drug RT predicts prolonged therapeutic effect(s), prolonged drug RT analysis targeting BLVRB inhibitors may have greater relevance as a negative phenotype predictor. Conclusions are limited, however, by the tight clustering of inhibitor IC_50_s (Table S4), and the single inhibitor displaying prolonged RT.

**Figure 6:**
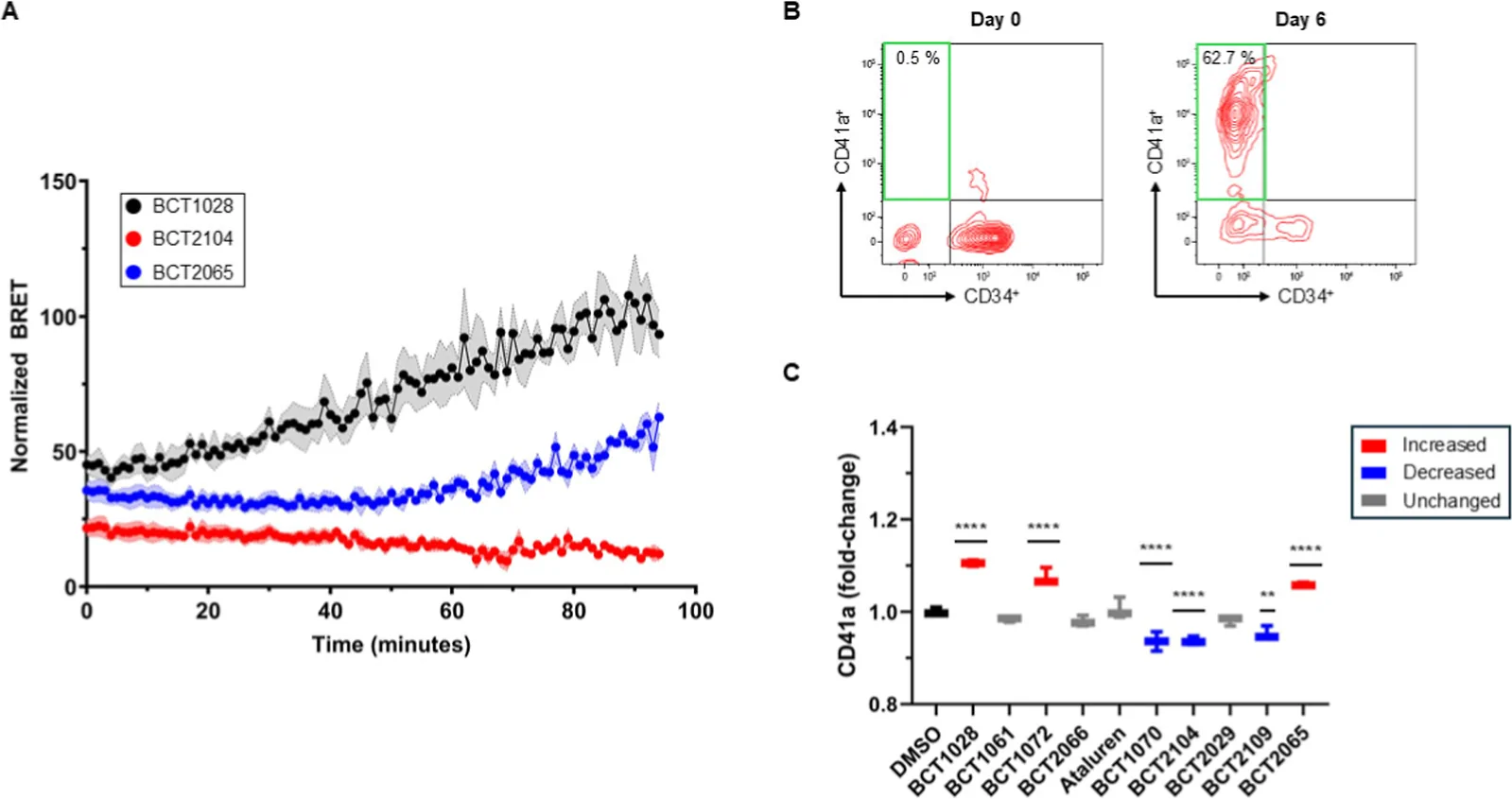
Drug residence time identifies differential binding kinetics and pharmacological modulation of megakaryocyte differentiation. **A.** HEL/BLVRB_NLuc cells (2 × 10^4^ cells/well) were preincubated with individual compounds at their respective IC_50_s for 120 min at 37 °C, followed by time-dependent affinity displacement using saturating BCT2101 (1 μM) for kinetic determinations. Shown are the normalized BRET determinations for representative compounds displaying short (BCT1028), intermediate (BCT2065), and prolonged (BCT2104) residence times. Data are mean ± SEM (shaded) from 3 independent experiments (see Table S4 for residence time determinations for all small molecules). **B, C.** CD34^+^ HSCs (1 × 10^4^/mL) were propagated in stem cell expansion media with thrombopoietin (TPO, 10 ng/mL) supplemented without (DMSO *v/v* control), or with 10 μM of individual compounds every 72 h, followed by flow cytometric detection of cell–surface CD41a (integrin α_IIB_; defined by green rectangle in scattergrams (*Panel B*); the mean control CD41a % at Days 0 and 6 is shown as a reference. *Panel C* box plots (interquartile range, median, and minimum/maximum data range) display the Day 6 CD41a fold-change normalized to (*v/v*) DMSO controls across the subset of *N* = 11 small molecule inhibitors (data are the mean ± SEM from *N* = 3 biological and technical replicates); *p*-value **<0.01, ****<0.0001 using unpaired *t*-test.

### BLVRB Affinity Ligands as Subcellular Tracers

Initial live cell imaging established the capacity of affinity ligands to perform as target-specific subcellular tracers in cells ectopically expressing BLVRB_NLuc (HEK293/BLVRB_NLuc). Confocal microscopy confirmed cellular penetration and predominant cytoplasmic accumulation of both ligands, although BCT2101 displayed a component of ancillary cell membrane localization. There was no evidence for nuclear localization using either probe (Figure S12B). Molar-excess unlabeled BCT2029 (20 μM) resulted in visual and statistically significant fluorescence loss of BCT2021 (p-value <0.001, Figure S12A,C) and BCT2102 (p-value <0.001) as demonstrated by quantitative image analyses (Figure S12B,D). Competitive displacement by unlabeled BCT2029 ligand *in vitro* recapitulated observations in rBRET assays, confirming cell-specific binding fidelity comparable to that seen using the biochemically pure recombinant system (Figure 4H, I, *vide supra*).

### BLVRB Active Site Engagement Spatially Partitions within the Endoplasmic Reticulum

We extended these studies to dissect the spatial and subcellular distribution(s) of BLVRB active site engagement, using naïve HEL cells known to express endogenous BLVRB.^19^ Similar to the patterns in HEK293/BLVRB_NLuc cells (*vide supra*), confocal microscopy demonstrated a cytoplasm-predominant pattern of BCT2101 fluorescence in HEL cells known to express BLVRB^19^ (Figure 6A). Given the critical role of the endoplasmic reticulum in regulating the cellular stress response and maintaining proteostasis, we applied live cell imaging to better define spatial and ER-specific distribution of endogenous BLVRB. For these experiments, HEL cells were dual-labeled with BCT2101 and the ER-selective fluorophore (glibenclamide_BODIPY^FL^), a fluorescently labeled derivative that selectively binds sulfonylurea receptors (SURs) localized to the ER.^29^ Indeed, visually evident overlap between BCT2101 and glibenclamide_BODIPY^FL^ fluorescent signals was readily demonstrated by confocal microscopy (Figure 7A). Image analysis followed by quantitative colocalization metrics confirmed near-saturating distributions between BCT2101 and glibenclamide_BODIPY^FL^ (Pearson coefficient r = 0.92, Figure 7B), with highly significant p-values across the cellular (N = 94) data set (Figure 7C). We then applied intensity thresholding to determine the Manders^30^ colocalization coefficients for BCT2101 (M1) and glibenclamide_BODIPY^FL^ (M2). The magnitude of the colocalization(s) was somewhat lower but remained highly correlated (M1 ∼ 0.84, M2 ∼ 0.85, Figure 7D), and presumably explained by regional loss of low-intensity colocalization signals.^30^ Nonetheless, these data suggest near-saturating colocalization of BCT2101 to the ER, with minimal unoccupied ER reserve. These observations suggested that preferential ER localization may regulate physicochemical properties or activation-dependent ER responses.

**Figure 7:**
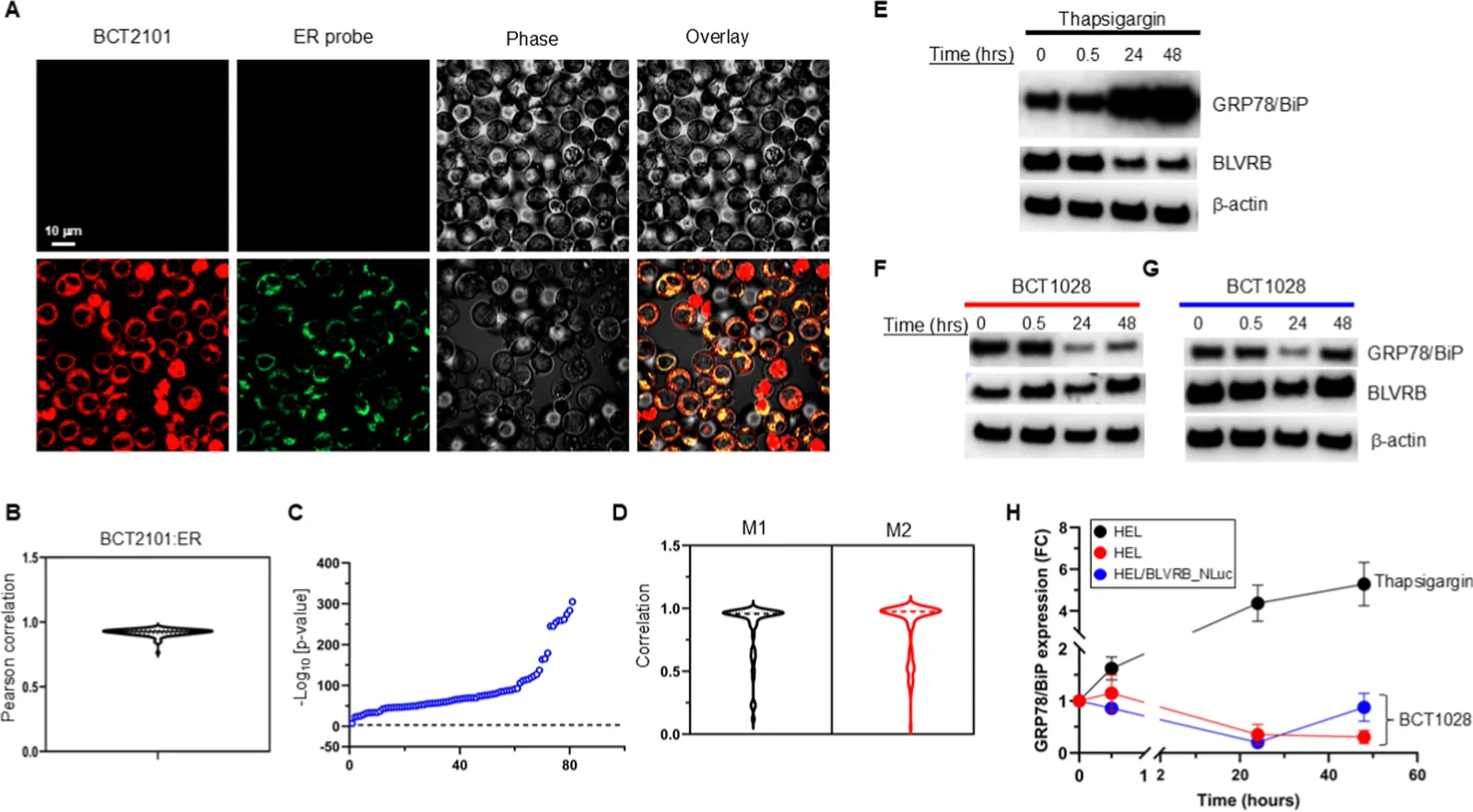
BLVRB affinity ligand reveals ER-predominant localization and transient modulation of GRP78/BiP with BLVRB inhibition. **A.** Human erythroleukemia (HEL) cells were incubated with BCT2101 (2 μM), the ER probe glibenclamide_BODIPY^FL^ (1 μM), or both probes, followed by washout, and live cell imaging by confocal microscopy. The dark images (left two *upper panels*) confirm the lack of fluorescence (specificity) of individual fluorophores using nonsynonymous excitation/emission spectra, while the overlay demonstrates the congruent yellow appearance resulting from overlap of red (BCT2101) and green (ER) pixels; size bar for all images is shown. **B, C.** Pearson correlation coefficients extracted from *N* = 94 cells were determined using pixel intensities from BCT2101 and glibenclamide_BODIPY^FL^ channels (*Panel B*); the corresponding p-values (−log_10_) are shown in *Panel C* dashed line corresponds to p-value 1 × 10^–3^ (−log_10_ + 3). **D.** The distributions of thresholded Manders (M1 and M2) coefficients calculated for *N* = 94 cells (mean ± SEM) were 0.84 ± 0.02 (M1) and 0.85 ± 0.02 (M2), establishing near-saturating colocalization of BCT2101 to the ER, and minimal unoccupied ER reserve. **E.** HEL cells (1 × 10^5^) were incubated with thapsigargin (1 μM), followed by immunoblot analyses (20 μg/lane). **F. G.** HEL (*Panel F*) or HEL/BLVRB_NLuc (*Panel G*) cells (1 × 10^5^/experiment) were incubated with BCT1028 (20 μM) for specified times, followed by immunoblot analyses (20 μg/lane). **H.** GRP78/BiP expression was quantified by ImageJ software (normalized to actin), and data expressed as time-dependent fold-change (FC) over baseline (time 0). Aggregated expression data from thapsigargin- or BCT1028-treated cells are displayed as the mean ± SEM from two independent biological experiments.

### ER-Compartmentalized BLVRB Regulates GRP78/BiP Expression

Co-localization of BLVRB within the endoplasmic reticulum prompted studies to further dissect BLVRB active site engagement as a direct initiator of the ER functional response. ER stress is mediated by three ER receptors (IRE1 [inositol-requiring enzyme 1], PERK [PKR-like ER kinase], and ATF6α [activating transcription factor 6α]), all of which are collectively bound to the master chaperone stress sensor glucose-regulated protein 78/binding immunoglobulin protein (GRP78/BiP). Dissociation of GRP78/BiP during ER stress results in receptor displacement and concomitant release of thiol isomerases that catalyze protein folding.^31^ Accordingly, we characterized BiP expression as a quantifiable determinant of ER stress, directly comparing BLVRB inhibition with that of thapsigargin (TG); the latter is a SERCA (sarco/endoplasmic reticulum calcium ATPase) pump inhibitor that induces the unfolded protein response and ER stress by disrupting ER Ca^2+^ homeostasis.^32^ Expectedly, TG-stimulated HEL cells demonstrated striking (∼6-fold) and persistent BiP induction, evident at 24 h and extending to 48 h (Figure 7E). These patterns were in sharp contrast to GRP78/BiP expression patterns seen with BLVRB inhibition in HEL cells, where incubation with the BLVRB inhibitor BCT1028 caused ∼3-fold BiP reduction at 24 h, with a trend for persistent GRP78/BiP downregulation extending to 48 h (Figure 7F). Identical patterns were recapitulated in HEL/BLVRB_NLuc cells which again demonstrated transient BiP reduction at 24 h, although return to baseline expression at 48 h was more evident (Figure 7G,H); the latter excludes apoptotic cell loss that may accompany reduced BiP levels,^33^ and confirms the transient nature of BLVRB inhibition as a GRP78/BiP modulator of ER proteostasis.

## Discussion and Conclusions

We have developed orthogonal nanoBRET donor–acceptor reagents that function as selective and quantitative chemical probes for BLVRB active-site engagement. Probe performance and specificity were first established in a biochemically reconstituted, cell-free recombinant nanoBRET (rBRET) format and subsequently translated to a cellular nanoBRET (cBRET) assay. We specifically used hematopoietic cell lines ectopically expressing BLVRB–NanoLuc which recapitulate key features of human erythroid and megakaryocytic (HEL) differentiation.^19,27^ Platform applicability allowed determination of equilibrium binding parameters for two chemically distinct classes of BLVRB active-site inhibitors and established highly reproducible pharmacological behavior across rBRET, cBRET, and enzymatic reductase assays.^19^ Statistically significant, triangulated orthogonal assay correlations (Figure 5F,G; R^2^ ∼0.9 with collective p-values ≤1.8 × 10^–7^) validate specificity of the affinity ligand(s) as selective nanoBRET probes for direct quantification of on-target engagement. Cell-specific affinity ligand binding was further confirmed by demonstrating (1) statistically greater immunofluorescence intensity in HEL/BLVRB_NLuc cells compared to HEL_C control cells (Figure 5C, *p* <0.0001), and (2) molar-excess quenching (by unlabeled BCT2029) of BCT2021 (p-value <0.001, Figure S12A,C) and BCT2102 (p-value <0.001, Figure S12B,D) immunofluorescence in HEL/BLVRB_NLuc cells by confocal microscopy and quantitative image analyses. These aggregate data highlight reproducibility and specificity of affinity ligand active site binding, and minimize the possibility of off-target (or spurious) cellular activity from the lipophilic BODIPY fluorophore.^34^ While most nanoBRET-based probe studies are conducted in HEK293 cells,^28^ our results demonstrate that the BLVRB probe pair performs robustly in lineage-relevant hematopoietic models, enabling context-dependent evaluation of ligand engagement that extends beyond generic cellular backgrounds.

Crystallographic and nanoBRET studies demonstrate that both diazabicyclic and pyrazolopyrimidinone derivatives converge on a conserved BLVRB active-site binding mode defined by π–π stacking with the NADP^+^ nicotinamide, salt-bridge formation with Arg^174^, and hydrogen bonding to His^153^.^19,35^ This shared binding architecture underlies the strong concordance observed between nanoBRET-derived target engagement measurements (recombinant and cellular) and enzymatic inhibition, authenticating fluorescent BCT2029-based analogues as high-fidelity chemical probes of native BLVRB engagement. The BLVRB/NADP^+^/BCT2029 trimolecular crystal structure has been previously reported,^19^ although we were unable to obtain crystal structures of BLVRB/BCT2029_BODIPY ligands. We determined ∼2 Å structures of BLVRB crystals which were soaked with BCT2101 and BCT2102, but did not observe any density in the active site corresponding to a BODIPY-labeled scaffold. It is likely that addition of BODIPY makes the molecule too large to soak into the crystal lattice. Nonetheless, *in silico* studies of *apo* BLVRB structure, cofactor-bound BLVRB structure, and BLVRB/NADP^+^/BCT2029 crystal structures revealed a stable and well-defined protein architecture, suggesting that the overall BLVRB fold is largely maintained across the different structural states. Detailed analysis of the binding site with the ternary BLVRB/NADP^+^/BCT2029 complex identified conformational adjustments involving Arg^174^ and Phe^113^ with the aromatic ring of the benzoic acid moiety of BCT2029, interactions predicted to contribute to the stabilization of the small molecule within the binding site for productive ligand recognition.

The small molecule inhibitor panel examined here—while structurally diverse—exhibited inconsistent effects on megakaryocyte differentiation. Previous data have identified a ROS-regulated mechanism of BLVRB-induced megakaryocyte expansion using CD34^+^ hematopoietic stem cells, *Blvrb*-deficient mice, and mice treated with BLVRB inhibitors.^2,17,19^ The developmentally regulated ROS signal serves to prime hematopoietic progenitors for differentiation,^18^ and suggests a mechanism whereby a “ROS threshold” provides the signal for MK differentiation. Cellular intracellular ROS accumulation requires fastidious regulation as excess ROS surpassing cellular antioxidant function would result in cell death. Interestingly, MK BLVRB expression occurs at an early (Day 3–Day 4) time point during MK differentiation,^17^ suggesting that its MK-promoting effects are restricted to a discrete postcommitment phase of megakaryocyte development from bifurcated megakaryocyte/erythroid progenitor cells.^36^ Importantly, BLVRB small molecule inhibitors that promote MK differentiation remain functionally insufficient in the absence of TPO supplementation, confirming that BLVRB inhibition synergizes with the TPO/c-MPL signaling axis that is regulated by rapid intracellular JAK2 phosphorylation and divergent downstream effector pathways.^17,19^ These complex relationships may explain the limited predictability of either equilibrium binding (IC_50_) or residence time as sole determinants of megakaryocytopoiesis using our *in vitro* CD34^+^ screen (Figure 6). Firm conclusions are limited, however, by the relatively narrow range of binding affinities and residence times of small molecule inhibitors studied. Although recent data have implicated BLVRB as an *S*-nitrosylated intermediary critical for insulin receptor signaling,^13^ a comparable mechanism of BLVRB-dependent c-MPL modulating activity remains to be established. Predicated on known redox inhibitory characteristics, ligand binding modes would not predict any effects on BLVRB *S*-nitrosylation. Finally, an ancillary effect on ER stress-associated megakaryocytopoiesis also remains plausible (*vide infra*).

The duration of target engagement provides synergistic information relevant to biologic effects (extending beyond determinations of equilibrium binding).^28,37^ Although residence time analysis is frequently applied for kinase and cancer drug screens,^28,38^ or G-protein coupled receptor signaling^37^ as a predictive parameter of phenotypic response, applicability to hematopoietic differentiation models is less well-developed. Indeed, our studies suggest deleterious effects in settings of prolonged exposure (as demonstrated for BCT2104). The extended residence time of BCT2104 is putatively regulated by slowly reversible stabilizing interactions, including hydrogen-bonding opportunities introduced by the disubstituted urea and *meta*-methoxy substituents. These features may transiently engage proximal residues Gln^14^ and Asn^79^, the latter positioned to interact directly with the urea moiety, thereby increasing the energetic barrier to ligand dissociation (Figures 3E and S9). Interestingly, BLVRB has recently been identified as a unique breast cancer target,^39^ a situation where prolonged antiproliferative exposure may have greater relevance for ligands with enhanced target engagement *in vivo*. Expanded chemical libraries designed to orthogonally vary residence time, inhibitor activity, and phenotypic screens will be critical to more rigorously define structure–engagement–phenotype relationships and mechanism(s) relevant to megakaryocyte differentiation pathways.

Integration of the nanoBRET target-engagement platform with affinity-ligand colocalization studies in cells expressing endogenous BLVRB provides presumptive evidence linking BLVRB active-site engagement to modulation of the endoplasmic reticulum (ER) stress response. Indeed, these findings offer a mechanistic framework that contextualizes prior *in vivo* observations demonstrating that genetic ablation of *Blvrb* or pharmacological inhibition in murine models produces redox-dependent hematopoietic phenotypes that emerge selectively during poststress recovery.^2,19^ Computational colocalization analyses applying the Manders coefficient established near-saturating localization of BCT2101 to the ER, results consistent with those demonstrating BLVRB antigenic colocalization with ER-localized heme oxygenase in breast cancer.^39^ Although ER colocalization of BLVRB active site engagement was near saturating, the molecular determinants underlying ER targeting remain unresolved, presumably explained by BLVRB anchoring through protein–protein interactions, lipid microdomains, or dynamic recruitment during stress. Dissection of these various mechanisms remains ongoing, but is relevant for thrombopoiesis since the ER establishes contact sites within the demarcation membrane system (DMS) that is necessary for lipid/membrane transfer during the final stages of megakaryocytopoiesis and proplatelet formation.^40^ These studies may also provide insight into how ER-localized BLVRB integrates redox control with stress signaling to effect lineage fate determination during erythropoiesis.^41^

Selective inhibition of the BLVRB active site using BCT1028 uncovers a regulatory role in ER redox buffering that is mechanistically distinct from canonical unfolded protein response (UPR) induction,^42^ a characteristic of megakaryocyte maturation proposed as an adaptive survival mechanism.^43^ Whereas the canonical ER stressor thapsigargin robustly induces GRP78/BiP expression through transcriptional and translational upregulation,^44^ BLVRB inhibition elicited a distinct response characterized by a transient, time-dependent reduction in GRP78/BiP levels at 24 h, followed by variable recovery trends at 48 h in two distinct BLVRB-expressing cell lines (Figure 7F–H). This pattern is inconsistent with classical UPR activation, but supports a model in which BLVRB modulates basal ER proteostasis by maintaining local redox homeostasis rather than directly triggering stress signaling. GRP78/BiP functions as the master chaperone and primary upstream sensor for the three stress receptors (IRE1, PERK, and ATF6α) to maintain their inactivity. Attenuated expression (as evident with BLVRB inhibition) would lower the threshold for UPR activation, resulting in cells predicted to display enhanced stress sensitivity without triggering canonical UPR signaling. These findings suggest that basal BLVRB activity preserves local ER redox homeostasis and GRP78/BiP buffering capacity (possibly by altered NAD­(P)­H/NADP^+^ ratios^39^), effectively decoupling redox regulation from overt UPR induction. Pharmacological disruption of this buffering system would possibly shift the stress activation threshold, sensitizing cells to ER perturbation while avoiding constitutive stress signaling. Within this framework, BLVRB hypothetically functions as an ER localized redox sensor^44,45^ whose biological output is determined by spatiotemporal active site engagement rather than equilibrium inhibition alone. Although our initial studies focused on GRP78/BiP as a primary readout for ER proteostasis,^46^ more robust conclusions require dissection of downstream effectors (IRE1, PERK, and ATF6α), thiol isomerase activity and selective UPR branch activation. While unexplored, these studies remain mechanistically justified based on our initial observations.

## Experimental Methods

### Ethics Statement

All research completed in this body of work complies with federal guidelines pertaining to ethical regulations and biosafety. Human Subjects studies for isolation and use of human hematopoietic stem cells from umbilical cords were approved by the Stony Brook University Institutional Review Board (IRB), human subjects assurance FWA #00000125; umbilical cords were obtained after written informed consent, and anonymized data were reported for analyses.

### Materials

FMN (flavin mononucleotide), NADPH (nicotinamide adenine dinucleotide phosphate), DCPIP (2,6-dichlorophenolindophenol), and HEPES (4-(2-hydroxyethyl)-1-piperazineethanesulfonic acid) were purchased from Sigma.

### Protein Expression and Purification

Codon-optimized nucleotide sequences encompassing BLVRB_NLuc, NLuc_BLVRB, or NLuc were synthesized by Genecopoeia (Rockville, MD), and assembled in pET-28 or pET-29 expression plasmids. BLVRB residues encompassed amino acids 1–206 (full-length, UniProt ID P30043), while nanoluciferase sequences encompassed catalytic domain residues 26–196 (UniProt ID Q9GV45). *E. coli* Rosetta (DE3) cells harboring individual plasmids were grown in LB medium containing 30 μg mL^–1^ kanamycin and 34 μg mL^–1^ chloramphenicol overnight at 37 °C. Overnight cultures were expanded in 500 mL volumes, grown at 37 °C to mid log phase (OD_600 nm_ ∼ 0.7), and protein production was induced using 0.2 mM IPTG for 16 h. Cells were harvested by centrifugation at 10,000 × *g* for 20 min at 4 °C, resuspended and sonicated in NP buffer (50 mM Tris–HCl (pH 8.0), 300 mM NaCl, 10 mM Imidazole) supplemented with 1 mM phenylmethylsulfonyl fluoride (PMSF), and cellular debris was removed by centrifugation as previously described.^47^ The cleared lysate was loaded onto a Ni-NTA resin column equilibrated in native binding buffer (60 mM NaH_2_PO_4_, pH 8.0, 500 mM NaCl) overnight at 4 °C, after which the column was washed with the binding buffer and proteins were eluted using a stepwise gradient of binding buffer containing 50 mM to 250 mM imidazole. The eluted fractions were analyzed by SDS-PAGE and those containing pure protein were pooled and dialyzed against 100 × volume of PBS at 4 °C overnight, followed by concentration and further removal of imidazole using a 3000 MWCO (molecular weight cutoff) centrifugal concentrator (Vivaspin 20, Sartorius); purity was >95% as established by SDS-PAGE and densitography.

Recombinant BLVRB used for enzymatic studies was expressed and purified as a glutathione *S*-transferase (GST) fusion protein in BL21­(DE3) cells.^10,17,20^ Briefly, protein expression was induced using 0.1 mm isopropyl β-d-1-thiogalactopyranoside and allowed to proceed for 3 h at 37 °C. GST-tagged BLVRB was purified by affinity chromatography using glutathione-Sepharose 4B, and the fusion protein was cleaved overnight at 4 °C with 1 nM α-thrombin, resulting in >95% separation from the GST carrier. The cleaved protein was repurified by a final gel filtration step on Sephacryl S-200, and was >95% pure as established by SDS-PAGE and densitography. Protein concentration of all recombinant proteins was determined using the bicinchoninic acid assay^10,48^ with BSA as standard, and purified proteins were aliquoted and stored at −80 °C.

#### Biochemical Assays

BLVRB (or BLVRB/nanoluciferase chimers) activity assays were performed with a Cary 60-UV visible spectrophotometer, using 2,6-Dichlorophenolindophenol (DCPIP) and flavin mononucleotide (FMN) as substrates. DCPIP reduction proceeded in a reaction containing 100 mM HEPES, pH 7.0, 100 μM NADPH, dose-modified inhibitors, and 50 μM DCPIP at 25 °C. Reactions were initiated by the addition of 100 nM BLVRB (or BLVRB/nanoluciferase chimers) and reductase activity was measured spectrophotometrically by following the decrease in absorbance at 600 nm corresponding to the reduction of DCPIP (ε = 20.7 mM^–1^ cm^–1^).^49^ Assays lacking inhibitor (solvent only) and/or enzyme served as positive and negative controls, respectively. Flavin reductase activity was determined by monitoring the rate of oxidation of NADPH to NADP^+^ at 340 nm (ε = 6200 M^–1^ cm^–1^). Enzymatic activity was measured at 25 °C in 100 mM HEPES, pH 7.0, containing 200 μM FMN, 100 μM NADPH, and varying concentrations of inhibitor. The concentration of inhibitor required to inhibit enzymatic activity by 50% (IC_50_) was determined using 8-point screens concentrations (0–5000 nM) in reaction mixtures at fixed concentrations of substrate (200 μM FMN or 50 μM DCPIP) and NADPH (100 μM). The total amount of organic solvent added was kept constant at a final concentration of 2% (v/v). IC_50_ values were determined by nonlinear regression fits using GraphPad Prism (version 9.4.0).

### Computational Modeling

#### In silico Docking

##### Input Preparation

The 3D structures of BCT2101 (BCT2029 coupled with a PEG linker and the BODIPY fluorophore) and BCT2102 (BCT2029 coupled with the BODIPY fluorophore) were prepared with the Schrödinger LigPrep module (Schrödinger Release 2024–3: LigPrep, Schrödinger, LLC, New York, NY, 2024). Standard options for tautomeric and protonation states were applied. For the protein models, the structure of BLVRB cocrystallized with the BLVRB inhibitor BCT2029 and NADP^+^ (UniProt code: P30043; PDB ID 5OOH;^25^ residues 1–206) and the crystal structure of NLuc (luciferase; UniProt code: Q9GV45; PDB ID 5IBO; catalytic domain residues 26–196) in its open β-barrel conformation^25^ were used for *in silico* modeling. For the latter, a monomeric assembly was considered as per experimental data.^25^ In the case of BLVRB, water molecules required to preserve protein–ligand interactions were retained during modeling. The Schrödinger Protein Preparation Tool^50^ was used to prepare all the protein structures (standard options).

##### Protein–protein (P–P) Docking

PyDock^51^ was used to generate the putative BLVRB-NLuc and NLuc-BLVRB chimers. A residue-based constraint (BLVRB residues: Asn^79^, Arg^78^, Asp^156^, Gln^157^, Pro^158^, Arg,^34^ Ser,^37^ Gly^50^ and Gln^57^) was also applied to focus the search near the inhibitor binding site at the BLVRB C-terminal and N-terminal, respectively. An additional proximity constraint (C–N atom distance <0.8 nm) between the C- and N-termini of the BLVRB_NLuc and NLuc_BLVRB chimeras was also applied to ensure the generation of plausible 3-dimensional geometries consistent with the experimental constructs. Up to 10,000 *in silico* models *per* system were generated and top 100 best ranked/scored protein–protein complexes were stored and analyzed. Chimer models having a protein–protein assembly compatible with the binding of the compounds of interest and maintaining structural consistency with the C- and N-terminal connections in both chimeras, were finally considered for ligand docking.

##### Ligand Docking

Up to 10 chimer models for BLVRB-NLuc (or NLuc-BLVRB) generated by pyDock^51^ were considered to explore the putative binding mode of BCT2101 and BCT2102. The X-ray defined compound (BCT2029) was used to center the docking grid and a radius of 35 Å, also covering the putative binding surface of the fluorophore moiety on NLuc, was considered. The Schrödinger Glide software (Standard Precision protocol, standard options) was used to perform the docking. A H-bond constraint on the BLVRB residue, Arg^174^ (involved in a salt bridge interaction with the acidic moiety of BCT2029) was also applied during docking to ensure a proper positioning of the two modeled compounds, BCT2101 and BCT2102. Up to 20 docking poses *per* ligand were generated and best models/solutions were then selected according to the docking scores (NLuc_BLVRB­[BCT2101/BCT2102]: [−11.19/–11.25] kcal/mol; BLVRB_NLuc-[BCT2101/BCT2102]: [−11.90/–10.92] kcal/mol) and visual inspection. The generated complexes were then energy minimized by using the minimization tool (standard options) implemented in the Schrödinger Prime module (Schrödinger Release 2024–2: Prime, Schrödinger, LLC, New York, NY, 2024). Final figures were prepared with PyMOL (version 3.0.4, the PyMOL Molecular Graphics System, Schrödinger, LLC).

### BLVRB Cocrystallization

BLVRB/NADP + crystals were grown at 18 °C with sitting drop vapor diffusion. BLVRB (17.8 mg/mL) was incubated with 1 mM NADP^+^ on ice prior to crystallization. Drops comprised 0.5 μL BLVRB/NADP^+^ and 0.5 μL well solution [0.1 M MES monohydrate (pH 6.5), 12% *w/v* PEG 20,000, and 25% (*v/v*) MPD (2-methyl-2,4 -pentanediol)]. Ligand cocrystals were prepared by soaking BLVRB/NADP^+^ for 20 h in a drop containing 2 mM BCT2066, BCT2104, or BCT2109. Crystals were cryoprotected with 20% glycerol before plunge freezing.

#### Data Collection and Processing

X-ray diffraction data were collected for BLVRB/NADP^+^, BLVRB/NADP^+^/BCT2066, BLVRB/NADP^+^/BCT2104, and BLVRB/NADP^+^/BCT2109 at the AMX beamline (17-ID-1) at the National Synchrotron Light Source II at Brookhaven National Laboratory (Upton, NY). In each case complete data sets were collected from single crystals. Each data set comprised a 200° rotation with 0.1° per frame and an exposure time of 0.005 s using radiation at a wavelength of 0.9201 Å. Diffraction data were indexed, integrated, and scaled with XDS,^52^ followed by merging with AIMLESS,^53^ iteratively performed with the autoPROC processing pipeline.^54^ The data collection and processing statistics are summarized in Table S1; raw diffraction data were deposited with Zenodo.

##### Structure Solution and Refinement

Trimolecular BLVRB/NADP^+^/compound (BCT2066, BCT2104, BCT2109) phases were determined via molecular replacement with *Phaser* ^55^ using the coordinates of BLVRB (PDB entry 5OOH)^24^ stripped from ligands and solvent as search model. The resulting models were subjected to alternating cycles of manual building with COOT^56^ and refinement with PHENIX^57^ until model completion. Ligand restraints were generated with *PHENIX eLBOW* using AM1 optimization and SMILES input. BLVRB/NADP^+^/BCT2066, BLVRB/NADP^+^/BCT2104, and BLVRB/NADP^+^/BCT2109 were refined to final Rwork/Rfree values of 0.173/0.207, 0.195/0.221, and 0.208/0.245, respectively. The refined three-dimensional coordinates and structure factors were deposited at the Protein Data Bank with accession codes 13EA (BCT2066) and 13DZ (BCT2104), and 13EB (BCT2109). Molecular structures shown in Figure 3 were depicted with PyMOL, *version* 1.6.0 (Schrödinger). Authors will release the atomic coordinates upon article publication.

## Medicinal Chemistry

**BCT2101** [3-(2-{N-14-[({m-[5-(*m*-carboxyphenyl)-3-ethyl-7-oxo-1,4,7a-triaza-2- indenyl]­phenyl}­methyl)­carbamoyl]-3,6,9,12-tetraoxatetradecylcarbamoyl}­ethyl)-4,4-difluoro-5-(2-pyrrolyl)-3a,4-dihydro-3aλ, 4adiaza-4λ -bora-s-indacen-4a-ium-3a-ide].

BCT2101 was synthesized in a six-step procedure (refer to Figure 1): Sulfuric acid (H_2_SO_4_, 5 mL) was added to *m*-cyanobenzoic acid (**1**, 204 mmol) in ethanol (0.3 L) and stirred overnight at 85 °C. The reaction mixture was concentrated *in vacuo*, mixed with NaHCO_3_ solution, extracted with ethyl acetate (3 × 200 mL), and washed with water and brine solution. The organic layer was dried over sodium sulfate and concentrated *in vacuo* as ethyl *m*-cyanobenzoate (23 g, 64.39%). Then, a solution of butyronitrile (1.6 eq, 183 mmol), tetrahydrofuran (THF, 0.16 L, 4.91 mol), and lithium 1-butanide (*n*-BuLi, 1.6 eq, 183 mmol) was stirred for 30 min at −78 °C, after which ethyl *m*-cyanobenzoate (114 mmol in THF) was added dropwise for 1 h. The reaction mixture was quenched with NH_4_Cl, extracted with ethyl acetate, dried over Na_2_SO_4_, reduced under vacuum, and purified by combi-flash chromatography and eluted with ethyl acetate in hexane. Collected pure fractions were concentrated under reduced pressure to 2-(*m*-cyanobenzoyl)­butryonitrile. Then, diazane (5 eq, 261 mmol) was added to 2-(*m*-cyanobenzoyl)­butryonitrile (52.2 mmol) in methanol and reflux reaction mixture at 80 °C for 16 h, whereupon the mixture was concentrated to dryness, made as an azeotrope with toluene and concentrated under vacuum to m-(5-amino-4-ethyl-3-pyrazolyl)­benzonitrile. To this solution (39.9 mmol) in THF was added lithium alumanuide (LAH, 3 eq, 120 mmol, 1 M in THF) at 0 °C and stirred at RT for 24 h. The reaction mixture was quenched with saturated NH_4_Cl, filtered through a Celite bed, concentrated under vacuum, dissolved in 5% MeOH in dichloromethane (DCM), and filtered multiple times through a Buchner funnel lined with filter paper to produce 3-[m-(aminomethyl)­phenyl]-4-ethyl-5-pyrazolamine (**2**, 27.7 mmol). Ethyl (*m*-methoxycarbonylbenzoyl)­acetate (1.1 eq, 30.5 mmol) was added to **(2)** in acetic acid and stirred at 150 °C for 5 h, cooled to 25 °C, filtered and washed with ethyl acetate to produce methyl m­[2-(*m*-cyanophenyl)-3-ethyl-7oxo-1,4,7a-triaza-5-indenyl]­benzoate **(3)**; (**3,** 246 μmol) was added to a stirred solution of 15-[(*tert*-butyl)­(oxycarbonylamino)]-4,7,10,13-tetraoxapentadecanoic acid (DIPEA, 246 μmol) in dimethylformamide (DMF) and 1,1,3,3-tetramethyl-2-(3H-1,2,3,4-tetraazainden-3-yl)-3-isoureaium hexafluoridophosphate­(1-)­(HATU) at 0 °C, followed by N-ethylbis­(isopropryl)­amine, and stirred at 25 °C for 16 h. Water was added and the organic layer extracted with 10% MeOH in DCM, dried over Na_2_SO_4_ in vacuum, and purified with column chromatography using 4–6% MeOH in DCM to produce methyl m-{2-[m-({14-[(*tert*-butyl)­(oxycarbonylamino)]-3,6,9,12-tetraoxatetradecylcarbonylamino}­methyl)­phenyl]-3-ethyl-7-oxo-1,4,7a-triaza-5-indenyl}­benzoate **(4)**. Lithium hydroxide (LiOH, 5 eq, 392 μmol) was added to **(4,** 78.4 μmol) in methanol and water, stirred at 25 °C for 16 h, concentrated *in vacuo*, diluted with water, and the organic layer was extracted with diethyl ether. The aqueous layer was acidified to pH ∼3 with 10% KHSO_4_ and the organic layer extracted with 10% MeOH in DCM, and dried over Na_2_SO_4_ in vacuum to produce m-{2-[m-({14-[(*tert*-butyl)­(oxycarbonylamino)]-3,6,9,12- tetraoxatetradecylcarbonylamino}­methyl)­phenyl]-3-ethyl-7-oxo-1,4,7a-triaza-5-indenyl}­benzoic acid **(5)**. HCl (685 μmol, 10 equiv) in 4 M dioxane was added at 0 °C to **(5**, 68.5 μmol) in DCM, stirred for 2 h, and concentrated in vacuum to produce m-(2-{m-[(14-amino-3,6,9,12-tetraoxatetradecylcarbonylamino)­methyl]­phenyl-3-ethyl-7-oxo-1,4,7a-triaza-5-indenyl)­benzoic acid **(6)**. N-ethylbis­(isopropyl)­amine (5 eq, 365 μmol) was added to (**6**, 73 μmol) in DMF and stirred for 10 min. To this mixture, 5-[3-(2,5-dioxo-1- pyrrolidinyloxy)-3-oxopropyl]-4,4-difluoro-3-(2-pyrrolyl)-4H-3a,4a-diaza-4-bora-s-indacen-3a-ium-4-uide (BODIPY, 1.1 eq, 80.3 μmol) was added, and the mixture stirred for 1 h at 25 °C in the dark, with progress of the reaction monitored using LC/MS, and subsequently purified by Prep-HPLC using 0.1% AA in ACN and lyophilized. The total yield was 9 mg (12.58%). ^1^H NMR (400 MHz, DMSO-*d*_6_): δ 13.18 (bs, 1H), 11.43 (s, 1H), 8.47 (t, *J* = 6.00 Hz, 1H), 8.36 (s, 1H), 8.09 (s, 1H), 8.04 (t, *J* = 5.2 Hz, 1H), 7.65 (s, 2H), 7.61 (d, *J* = 8.00 Hz, 1H), 7.43 (t, *J* = 8.00 Hz, 2H), 7.36 (s, 1H), 7.33 (d, *J* = 4.4 Hz, 1H), 7.30 (d, *J* = 13.6 Hz, 2H), 7.14 (d, *J* = 4.8 Hz, 2H), 7.00 (d, *J* = 3.6 Hz, 2H), 6.33 (d, *J* = 3.6 Hz, 2H), 5.95 (s, 1H), 4.36 (d, *J* = 4.8 Hz, 2H), 3.63 (t, *J* = 6.4 Hz, 2H), 3.48–3.39 (bs, 16H), 3.22 (q, *J* = 5.6 Hz, 2H), 3.12 (t, *J* = 8.00 Hz, 2H), 2.86 (q, *J* = 7.2 Hz, 2H), 2.40 (t, *J* = 6.00 Hz, 2H), 1.14 (t, *J* = 6.8 Hz, 3H). LCMS (*m*/*z*): 946.00 [M + H]^+^.

**BCT2102** [*5-{2-[({m-[5-(m-carboxyphenyl)-3-ethyl-7-oxo-1,4,7a-triaza-2-indenyl]­phenyl}­methyl)­carbamoyl]­ethyl}-4,4-difluoro-3-(2-pyrrolyl)-4H-3a,4a-diaza-4-bora-s-indacen-3a-ium-4-uide*].

BCT2102 was synthesized as follows: N-ethylbis­(isopropyl)­amine (5 eq, 386 μmol) was added to **(3,** 77.2 μmol) in DMF (63.4 mmol) and stirred for 10 min. BODIPY (1.1 eq, 85 μmol) was added and the reaction continued in the dark for 30 min at 25 °C, with progress monitored by LC/MS. The crude mixture was concentrated under vacuum, subsequently purified by Prep-HPLC using 0.1% AA in ACN and lyophilized. The yield was 8 mg (14.81%). ^1^H NMR (400 MHz, DMSO-*d*_6_): δ 12.93–12.53 (bs, 1H), 11.39 (s, 1H), 8.57 (t, *J* = 6.00 Hz, 1H), 8.41 (s, 1H), 8.10 (s, 1H), 7.68 (s, 1H), 7.64 (d, *J* = 8.00 Hz, 1H), 7.46–7.41 (bs, 2H), 7.36 (s, 1H), 7.33 (d, *J* = 4.8 Hz, 2H), 7.26 (s, 1H), 7.16 (d, *J* = 4.8 Hz, 2H), 7.00 (d, *J* = 3.6 Hz, 1H), 6.54 (s, 2H), 6.35–6.29 (bs, 2H), 5.94 (s, 1H), 4.40 (d, *J* = 6 Hz, 2H), 3.19 (t, *J* = 4 Hz, 2H), 2.86 (q, *J* = 7.2 Hz, 2H), 2.61 (t, *J* = 8.00 Hz, 2H), 1.16 (t, *J* = 6.4 Hz, 3H). LCMS (*m*/*z*): 698.83 (M + H)^+^.

Structures of all compounds were confirmed by ^1^H NMR spectroscopy and LC/MS/MS, and all compounds were >95% pure as established by HPLC analysis (Figures S1–S6). Compounds were suspended in 100% DMSO and stored at −20 °C.

### Bioluminescence Resonance Energy Transfer (nanoBRET)

#### Recombinant nanoBret Assay

A modified version of the nanoBRET assay^22^ was used to directly quantify tracer binding and competitive displacement using purified recombinant BLVRB_nLuc chimers. BLVRB_NLuc, NLuc_BLVRB, and NLuc (10, 50, and 100 nM) were assayed at 25 °C in 96-well white-bottom microtiter plates in a buffer containing 100 mM HEPES (pH 7.4) and 17.1 nM NaCl in 100 μL volumes. Tracer saturation curves were generated using dose-modified BCT2101 or BCT2102 (range 2 nM to 10 μM, final DMSO 1% *v/v*), and all readings were protected from light during the entirety of the acquisition measurements. For nonspecific binding, tracer titrations were performed in the presence of unlabeled BCT2029 (5 μM), selected based on maximal displacement of tracer signal and minimal (<5%) effect on donor luminescence (nonspecific binding). For competition binding experiments using BLVRB small molecule inhibitors, BLVRB_NLuc (50 nM diluted in the identical assay buffer) was pre-equilibrated with a tracer concentration (650 nM BCT2102) selected near the K_d_ to optimize the dynamic range and competitive displacement sensitivity.^28^ Dose-adjusted test compounds in DMSO (1% *v/v*) were added for 30 min at 25 °C in the dark to reach equilibrium, followed by addition of the nanoluciferase substrate (furimazine; 10 μL of 1:10 diluted stock in PBS, Promega catalog #N112). Light emissions at 450 nm (BRET donor) and 605 nm (BRET acceptor) were sequentially recorded within 3 min using a multimode microplate reader (BioTek Synergy H1, Agilent Technologies), and raw BRET ratios were calculated by dividing the acceptor fluorescence (605 nm) by donor luminescence (450 nm) signals [reported as milli-BRET (mBRET) units (mBU)].^58^ Data were blank-corrected [protein, furimazine without tracer, and DMSO (*v/v*)], and normalized BRET (%) was calculated relative to tracer-only (100%) and no-tracer (0%) controls. Apparent dissociation constants (*K*_*d*_) were determined by fitting specific binding values (total minus nonspecific) to a one-site saturation model using GraphPad Prism. Half-maximal inhibitory concentrations (IC_50_) were calculated by fitting normalized BRET (%) values plotted as a function of test compound concentration to a four-parameter sigmoidal dose–response model (GraphPad Prism 10).^58^

#### Cellular nanoBRET Target Engagement Assay

Cell-based nanoBRET assays were performed in digitonin-permeabilized cells at 37 °C as previously described,^22^ using HEK293 and HEL stable cell lines ectopically expressing BLVRB_NLuc (HEL/BLVRB_NLuc, HEK293/BLVRB_NLuc); experiments were completed on cell lines with ≤5 passages, and evaluated in triplicate to ensure technical reproducibility. Briefly, cells (2 × 10^4^/100 μL well) were seeded into 96-well white plates (Greiner Bio-One; catalog no. 655098) for 24 h, washed, and the media replaced with Opti-MEM containing 50 μg/mL digitonin to facilitate probe access. Tracer isotherms were generated using serially diluted BCT2101 or BCT2102 (2 nM to 10 μM) in the presence or absence of 20 μM BCT2029 (final DMSO 1% *v/v*) for 2 h prior to BRET measurements and determination of apparent K_d_, as outlined above For competitive binding experiments using BLVRB-targeted compounds, cells were pre-equilibrated with BCT2101 (1 μM, chosen to maximize assay window while maintaining robust competition sensitivity), followed by addition of serially diluted test compounds (2 nM to 10 μM, in DMSO 1% *v/v*) for 2 h prior to BRET measurements (50 μL mixture of NLuc substrate furimazine and inhibitor diluted in Opti-MEM, Promega, catalog no. N2162). Light emissions at 450 nm (BRET donor) and 605 nm (BRET acceptor) were sequentially recorded within 3 min, and BRET values were normalized relative to wells treated with BCT2101 alone (% tracer-only signal = 100%), or no tracer (background = 0%).

Ligand dissociation kinetics were measured at 37 °C to determine compound residence times. HEL/BLVRB_NLuc cells (2 × 10^4^ cells per 100 μL) in Opti MEM were incubated with DMSO (vehicle control, 1% v/v) or test compounds at their respective IC_50_ concentrations (final DMSO 1% v/v) for 120 min at 37 °C to allow target occupancy and binding equilibrium. Cells were then centrifuged (200 × *g* for 10 min), washed once with prewarmed Opti MEM, and resuspended in fresh Opti MEM containing saturating BCT 2101 (1 μM) to prevent ligand rebinding, digitonin 20 μg/mL, and the NanoLuc detection reagent. BRET measurements were initiated immediately in white 96 well plates, and early time points exhibiting transient signal instability following permeabilization were excluded from analysis. The apparent dissociation rate constants (K_off_) were fit in GraphPad Prism by nonlinear regression of time dependent BRET signal recovery using a one phase exponential association model. Compound residence times were calculated as τ = 1/K_off_.

### Cell-Based Studies

#### Cell Lines

Cells were obtained from ATCC (American Type Culture Collection) and maintained at 37 °C and 5% CO_2_ in a humidified incubator. Human erythroleukemia (HEL) cells were grown in RPMI, while HEK293 cells were maintained in DMEM (Dulbecco’s modified Eagle’s medium) supplemented with 10% fetal calf serum and 1% penicillin–streptomycin; all studies were completed on cells passaged ≤5 times.

#### BLVRB/NLuc Lentivirus

Lentiviruses expressing BLVRB_NLuc (*Lv/BLVRB_NLuc*) or empty vector control (*Lv_control*) were used for transduction and generation of stable cell lines in HEK293T or HEL cells using MOIs (multiplicity of infections) of ∼5 and puromycin (0.2 μg/mL) selection.^17,59^ Lentiviruses were driven by the cytomegalovirus promoter, contained the puromycin-resistant cassette, and were generated as previously described;^17^ nontransduced cells supplemented with puromycin resulted in complete cell death. Vesicular stomatitis virus (VSV)-pseudotyped lentiviruses were generated in HEK293T cells, and concentrated stocks were titered in NIH-3T3 cells. Viral transduction efficiencies were comparable, with infectious unit titers of 3.1 × 10^7^/mL (Lv/BLVRB_NLuc) or 5.0 × 10^6^/mL (Lv_Control).

#### Flow Cytometry Detection of BCT2021 and BCT2022

HEK293T/BLVRB_NLuc cells were seeded in 12-well plates (1 × 10^5^ cell/well) and grown for 24 h at 37 °C, at which point the media was replaced with fresh DMEM supplemented with dose-adjusted BCT2101 or BCT2102 (final 1% DMSO v/v) for 2 h at 37 °C in the dark. Cells were then washed × 2 with PBS, resuspended at 4 °C in the dark and analyzed on a CytoFLEX flow cytometer (Beckman Coulter) equipped with a 561 nm yellow-green laser line and 585/42 nm bandpass filter capable of light detection between 564 and 606 nm. Flow cytometric quantification was completed by data acquisition of 10,000 events using logarithmic gain settings for light scatter and fluorescence detection; data were analyzed using Kaluza Flow Cytometry Analysis Software (Beckman Coulter; Brea, CA).

#### CD34^+^ Hematopoietic Stem Cells (HSCs)

Immunoselected CD34^+^ cells isolated from human umbilical cord blood (independent of sex) served as the source for megakaryocyte differentiation, and were >95% pure prior to differentiation.^17,59^ Suspension cultures were expanded overnight in StemSpan SFEM II medium containing StemSpan CC100 expansion supplement, and megakaryocyte differentiation was completed in serum-free StemSpan SFEM II medium plus StemSpan CC220 (containing stem cell factor (SCF), IL-6, IL-9 and TPO cytokines; concentrations proprietary), without (*v*/*v* DMSO as control) or with 10 μM small molecule inhibitors at 72 h intervals. TPO-deficient cultures maintained in StemSpan SFEM II medium supplemented with IL-6, IL-9 and SCF (10 ng/mL each) failed to differentiate. At baseline and individual time points, aliquots were counted or differentiation was monitored by flow cytometry gating on live 7-actinomycin D (7-AAD)-negative cells for immunophenotypic quantification of CD41a (integrin α_IIb_, MK) and/or CD34.

#### Immunoblotting Analysis

Cells were lysed in 1X RIPA buffer (Rockland Immunochemical; Pottstown, PA) containing protease inhibitor cocktail (Millipore Sigma cat#P8340) and phosphatase inhibitor cocktail (Millipore Sigma cat#524625), cellular debris was removed by centrifugation at 10,000 *g* for 15 min at 4 °C, and protein concentrations were determined by bicinchoninic acid (BCA) assay^48^ with bovine serum albumin (BSA) as standard. Protein lysates were size-fractionated by SDS-PAGE,^17^ transferred to polyvinylidene fluoride (PVDF) membranes, and immunodetection was completed using sheep *anti*-BLVRB antibody (1:1000; R&D Systems; Minneapolis, MN cat#AF6568) followed by donkey antisheep antibody (1:2000; Jackson ImmunoResearch; West Grove, PA; cat#713–035–147); antiactin antibody (1:1000; Millipore Sigma; Burlington, MA cat#MAB1501R) followed by goat antimouse antibody (1:2000; Jackson ImmunoResearch; West Grove, PA cat#115–036–072); mouse *anti*-GAPDH antibody (1:1000; Invitrogen; Carlsbad, CA cat#MA5–15,738) followed by goat antimouse antibody (1:5000; Jackson ImmunoResearch; West Grove, PA cat#115–036–072); rabbit *anti*-GRP78/BiP antibody (1:1000, Cell Signaling Technology, antibody #3183), followed by goat antirabbit IgG (1:1000, Cell Signaling Technology, antibody #7074). Immunodetection was completed by enhanced chemiluminescence using Luminata Forte Western HRP substrate (Millipore Sigma; Burlington, MA) and visualized using a C–DiGit blot scanner (LI-COR; Lincoln, NE). ImageJ software^60^ was used for all densitometry calculations.

### Confocal Microscopy and Image Analysis

Adherent HEK293/BLVRB_NLuc cells seeded onto poly-d-lysine coated glass coverslips (P35GC-0–10 C.S, MatTek Corporation) were propagated for 48 h at 37 °C in a humidified incubator, at which point the media was replaced with Opti-MEM I reduced serum medium without phenol red (11,058,021, Gibco, Thermo Fisher Scientific). Cells were then supplemented with BCT2101 or BCT2102 (various concentrations), and incubated in the dark at 37 °C for 60 min. To visualize competitive binding, BCT2101 or BCT2102 were loaded simultaneously with unlabeled BCT2029 (20 μM), gently washed twice with Opti-MEM buffer, and then supplemented with 10 μM DAPI (4′,6-diamidino-2-phenylindole, Thermo Fisher Scientific) for nuclear imaging. HEL cell labeling was completed on live cells incubated at 37 °C with BCT2101 (60 min, 2 μM), glibenclamide_BODIPY^FL^ (30 min, 1 μM) or both compounds for colocalization analyses. After 60 min, cells were washed into phenol red-free imaging medium, and transferred onto glass coverslips for image capture. Live cell imaging was performed using a ZEISS LSM 980 confocal microscope equipped with Airyscan 2. Fluorophores were imaged sequentially to minimize cross-spectral detection. Glibenclamide_BODIPY^FL^ was excited at 488 nm and emission was collected through a 500–550 nm band-pass filter, while BCT2101/BCT2102 were excited at 561 nm with emission collected through a 570–620 nm band-pass filter. Images (512 × 512 pixels) were acquired using a high numerical aperture Plan-Apochromat 63*X*/1.4 oil DIC M27 objective, and detector gains were held constant across conditions (typical ranges: 600–800 V for 488 nm detection and 650–850 V for 561 nm detection). Images were imported into ImageJ software for quantitative image analysis,^61^ and no postacquisition contrast enhancement or nonlinear intensity transformations were applied prior to analysis. Automated cellular segmentation was completed using Cellpose,^62^ and BIOP JACOP plugin was applied for colocalization statistics.^61^ For individual regions of interest, cellular colocalization analyses were quantified using raw pixel intensities, and applied for determination of the Pearson correlation coefficient or the thresholded Manders’ overlap coefficients (M1 and M2); the latter was determined after automatic thresholding to exclude the background signal. To assess the statistical significance of observed colocalization, Costes’ randomization analysis was performed to generate a null distribution of Pearson correlation values, followed by calculation of the corresponding p-values for correlated and anticorrelated distributions.^30^ Data aggregation and statistics were compiled across multiple images and experimental replicates, and colocalization data sets were analyzed across three independent replicates to avoid cross-compartment bias.

### Statistical Analyses

Statistical comparisons were completed using ANOVA or Student’s *t* tests (or their nonparametric counterparts if the normality assumptions were not met) at the significance level of *p* < 0.05. Simple linear regression was performed in GraphPad Prism (*version* 9.3.1) for correlation analyses.

## Supplementary Material



## Data Availability

The authors declare that the data supporting the findings of this study are available within the manuscript and its Supporting Information (SI) file.

## Notes

The Supporting Information (SI) file contains The Supporting Information is available free of charge at https://pubs.acs.org/doi/10.1021/acs.jmedchem.6c01466.

HRMS data for BCT2101 and BCT2102 (Figures S1–S6), *In silico* studies of BLVRB/NLuc chimers and BLVRB conformational changes (Figures S7, S8), Refined crystallographic features of BLVRB/NADP+/BCT2104 (Figure S9), Supportive rBRET and cBRET developmental studies (Figures S10, S11, S12), Crystallographic details and BLVRB/NLuc nucleotide sequences (Tables S1–S3, *.doc), Detailed characteristics of BLVRB small molecule inhibitors (Table S4, *.doc) (PDF)

## Notes

Protein data bank: The refined three-dimensional coordinates and crystallographic structures were deposited at the Protein Data Bank (PDB) with accession codes 13DZ (BCT2104), 13EA (BCT2066), and 13EB (BCT2109). Authors will release the atomic coordinates upon article publication. Small molecule BLVRB inhibitors and ligands described in this manuscript are available upon request to Dr. W. Bahou.

## Abbreviations

ATF6α: Activating Transcription Factor-6 alpha).
BiP: Binding immunoglobulin Protein).
BODIPY: Boron–dipyrromethene.
BRET: Bioluminescence Resonance Energy Transfer.
BR: Bilirubin.
BV: Biliverdin.
cBRET: Cellular Bioluminescence Resonance Energy Transfer.
cMPL: Cellular Myeloproliferative Leukemia protein (thrombopoietin receptor).
CD34: Cluster of differentiation marker 34 (early stem cell marker).
CD41a: Cluster of differentiation marker 41a (megakaryocyte integrin α_IIb_).
DCPIP: 2,6-dichlorophenolindophenol.
DIPEA: *N*,*N*-Diisopropylethylamine.
DR: DCPIP reduction activity.
ER: Endoplasmic reticulum.
FMN: Flavin mononucleotide.
FR: FMN reduction activity.
GRP78: Glucose-Regulated Protein 78.
GSSG: Glutathione disulfide, oxidized.
GSH: Glutathione, reduced;
HEK293: Human Embryonic Kidney 293 cells.
HEL: Human erythroleukemia cells.
HATU: Hexafluorophosphate Azabenzotriazole Tetramethyl Uronium.
HMOX1: Heme oxygenase 1.
HMOX2: Heme oxygenase 2.
HSC: Hematopoietic stem cell.
IRE1: Inositol-Requiring Enzyme 1.
LiOH: Lithium hydroxide.
mBU: Milli-BRET Units.
MeOH: Methanol.
MK: Megakaryocyte.
*n*-BuLi: *n*-Butyllithium.
NLuc: Nanoluciferase.
PERK: Protein kinase-like endoplasmic reticulum kinase.
PEG: Polyethylene glycol.
nPrCN: Propionitrile.
rBRET: Recombinant Bioluminescence Resonance Energy Transfer.
ROS: Reactive Oxygen Species.
RPMI: Roswell Park Memorial Institute 1640 defined media.
RT: Residence time.
TE: Target engagement.
TG: Thapsigargin.
TMR: Tetramethylrhodamine.
THF: Tetrahydrofuran.
TPO: Thrombopoietin.
UPR: Unfolded Protein Response.
