# Supplementary material for: A Structure-guided Active Site Affinity Ligand Unmasks a Stress-Sensitizing Role for BLVRB in the Endoplasmic Reticulum
Source: J Med Chem. 2026 Jul 27;69(16):19933–50. doi: 10.1021/acs.jmedchem.6c01466 (PMC13528197; doi:10.1021/acs.jmedchem.6c01466)
Supplement: Supplementary file 1 [file jm6c01466_si_001.pdf]

SUPPORTING INFORMATION

A structure-guided active site affinity ligand unmasks a stress-sensitizing role for BLVRB in the endoplasmic reticulum

Rahul Raghavan Thekke Veedu[1,2†], Jawaad Sheriff[1†], Natasha M. Nesbitt[1], Natalia Marchenko[1,3], Lisa Pennacchia[1], Tiziana Ginex[4], Patrick Hearing[5], Dale F. Kreitler[6], and Wadie F. Bahou[1,2,*]

[1]Blood Cell Technologies, JLABS@NYC, 101 6th Avenue, New York, NY 10013, USA

[2]Department of Medicine, Stony Brook University, Stony Brook, NY 11794, USA

[3]Deparment of Pathology, Stony Brook University, Stony Brook, NY 11794, USA

[4]Pharmacelera, Parc Científic de Barcelona (PCB), Baldiri Reixac 4-8, 08028 Barcelona, Spain

[5]Deparment of Microbiology and Immunology, Stony Brook University, Stony Brook, NY 11794, USA

[6]Center for Biomolecular Structure, Brookhaven National Laboratory, Upton, NY 11793, USA

†Rahul Raghavan Thekke Veedu and Jawaad Sheriff contributed equally to this work

<u>*Author Contact:</u>

Wadie F. Bahou, MD
Dept. Medicine, HSCT15-090
Stony Brook University
Telephone: 631-638-3885
Fax: 631-638-2727
Email: Wadie.Bahou@stonybrookmedicine.edu

TABLE OF CONTENTS

Cover page..**Page S1**

Table of Contents..**Page S2**

Figure S1. *^{1}H NMR (400 MHz) of BCT2101*..**Page.S3**

Figure S2. *HPLC chromatogram of BCT2101*..**Page S4**

Figure S3. *LC/MS/MS spectra of BCT2101*...**Page S5**

Figure S4. *^{1}H NMR (400 MHz) of BCT2102*..**Page S6**

Figure S5. *HPLC chromatogram of BCT2102*..**Page S7**

Figure S6. *LC/MS/MS spectra of BCT210*...**Page S8**

Figure S7. *Computational comparison of BLVRB_NLuc chimers*.................................**Page S9**

Figure S8. *Structural comparison of apo, cofactor-bound, and BLVRB/NADP$^+$/BCT2029 crystal structures*..**Page S10**

Figure S9. *BCT2104/NADP$^+$/BLVRB crystal structure*..**Page S11**

Figure S10. *Characterization of recombinant BRET target engagement assay.*.............**Page S12**

Figure S11. *Cellular nanoBRET characterization in HEK293/BLVRB_NLuc cells.*......**Page S13**

Figure S12. *Affinity ligand live cell imaging of HEK293/BLVRB_NLuc cells*...............**Page S14**

Table S1. *X-ray crystallographic data collection and refinement statistics*..............**Page S15**

Table S2. *BLVRB/nanoluciferase chimer sequences*..**Page S16**

Table S3. *Nanoluciferase/BLVRB chimer sequences*..**Page S17**

Table S4. *Characteristics of BLVRB small molecule inhibitors (N = 11)*..................**Page S18**

Figure S1

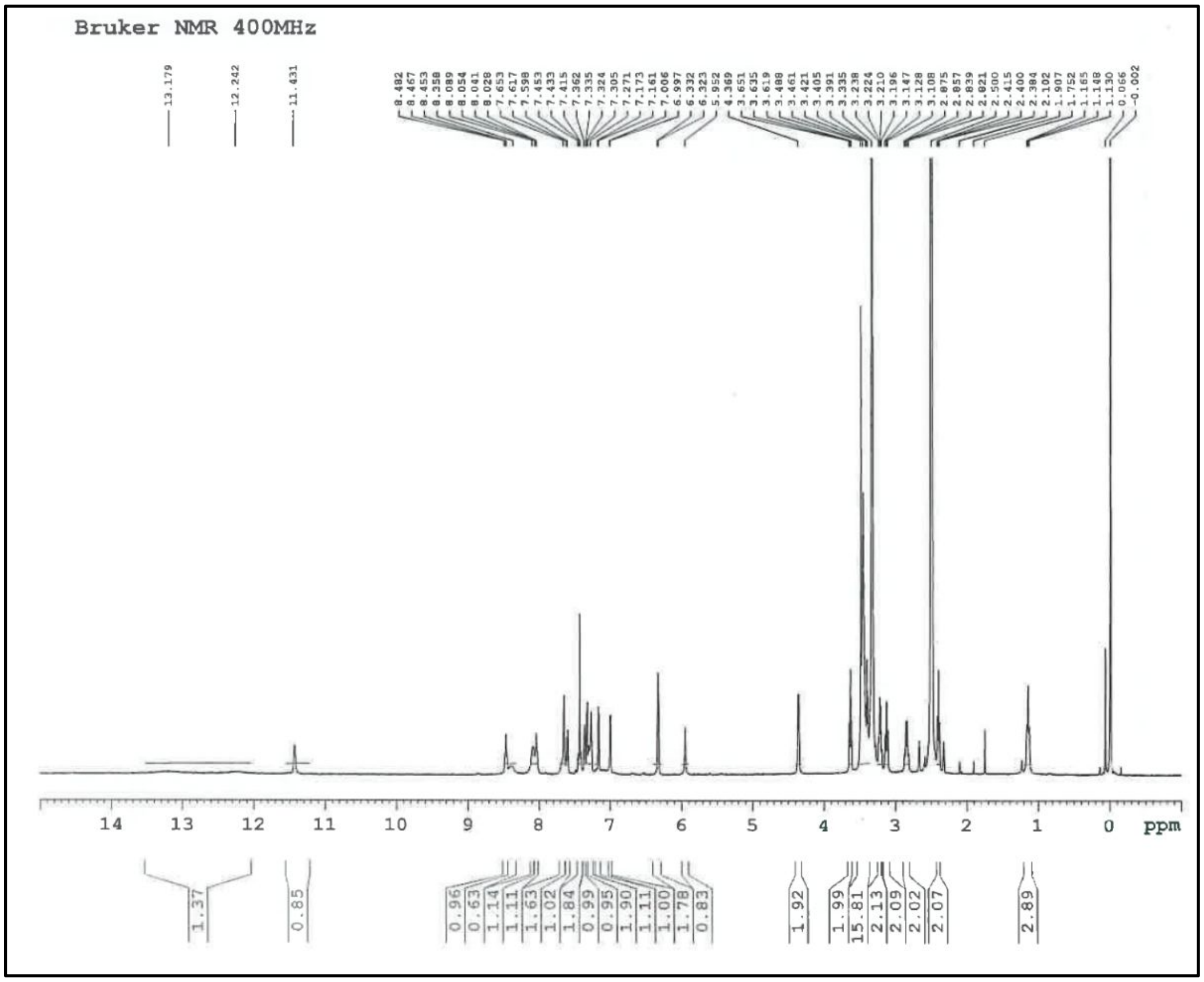

<u>Figure S1</u>. *^{1}H NMR (400 MHz) of BCT2101*

Figure S2

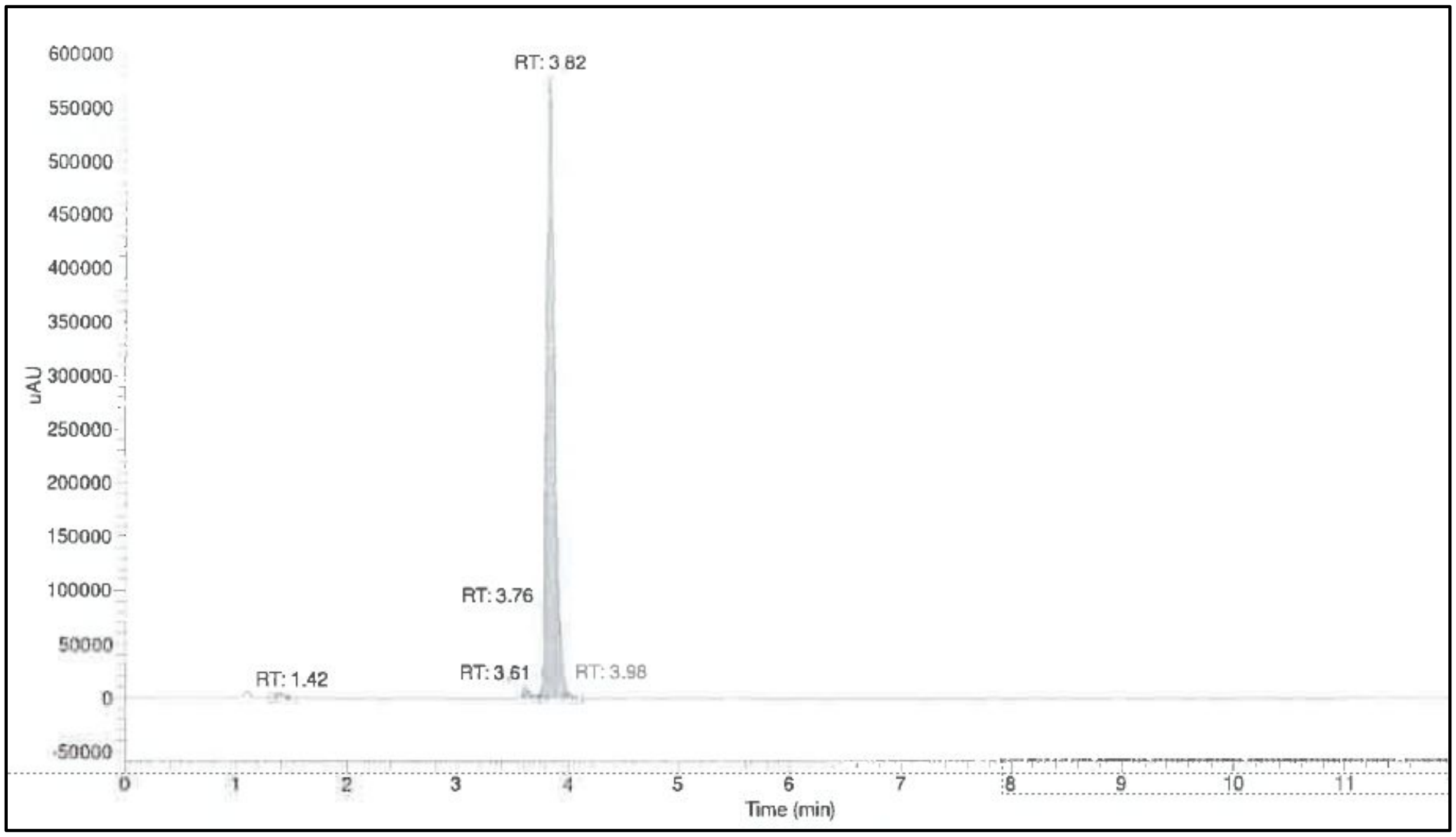

<u>Figure S2</u>. *HPLC chromatogram of BCT2101***.** Purity at peak retention time (3.82 minutes) is 95.10%.

Figure S3

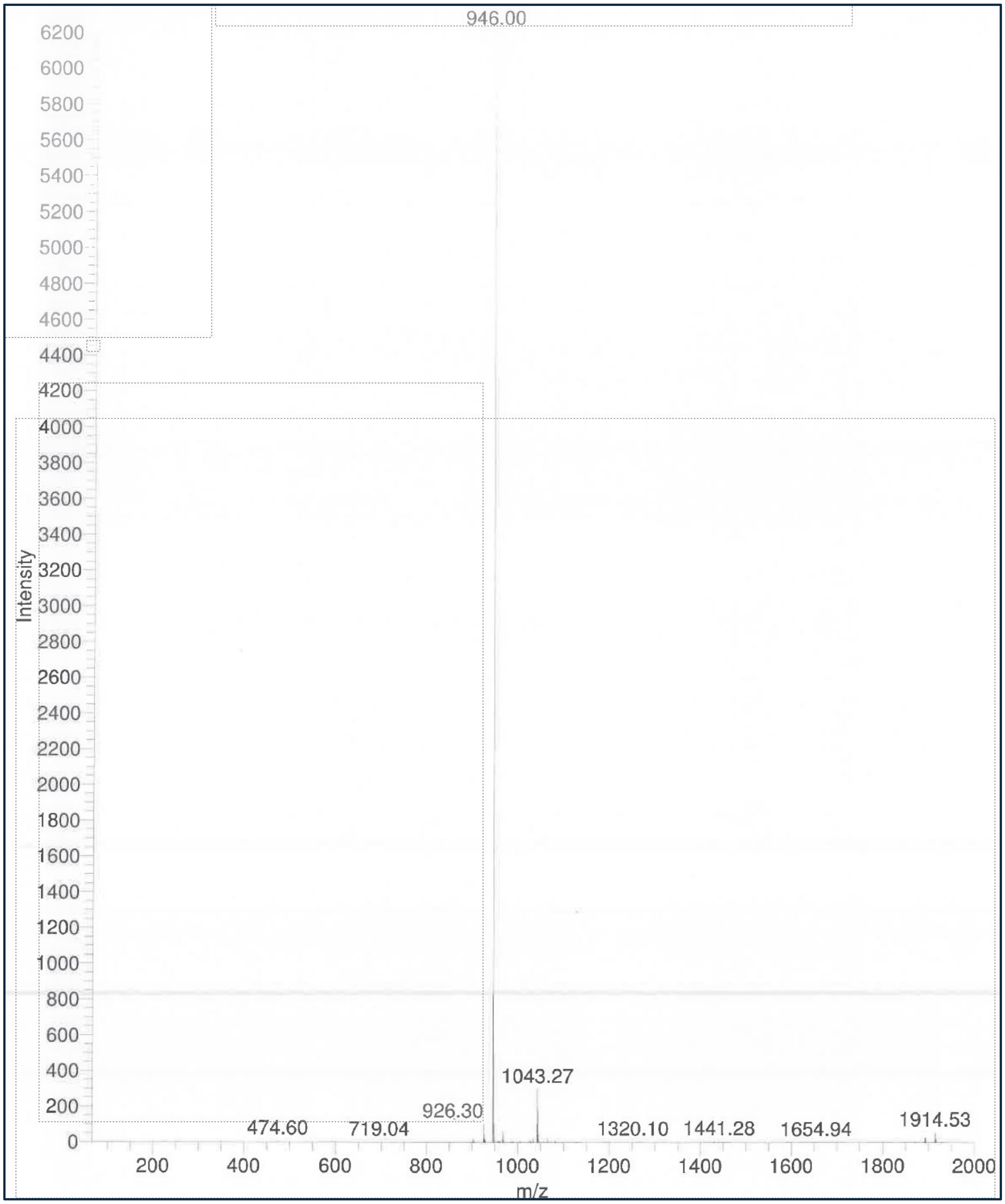

<u>Figure S3</u>. *LC/MS/MS spectra of BCT2101*. The predominant 946.00 $[M + H]^+$ peak is shown (96.62% purity).

Figure S4

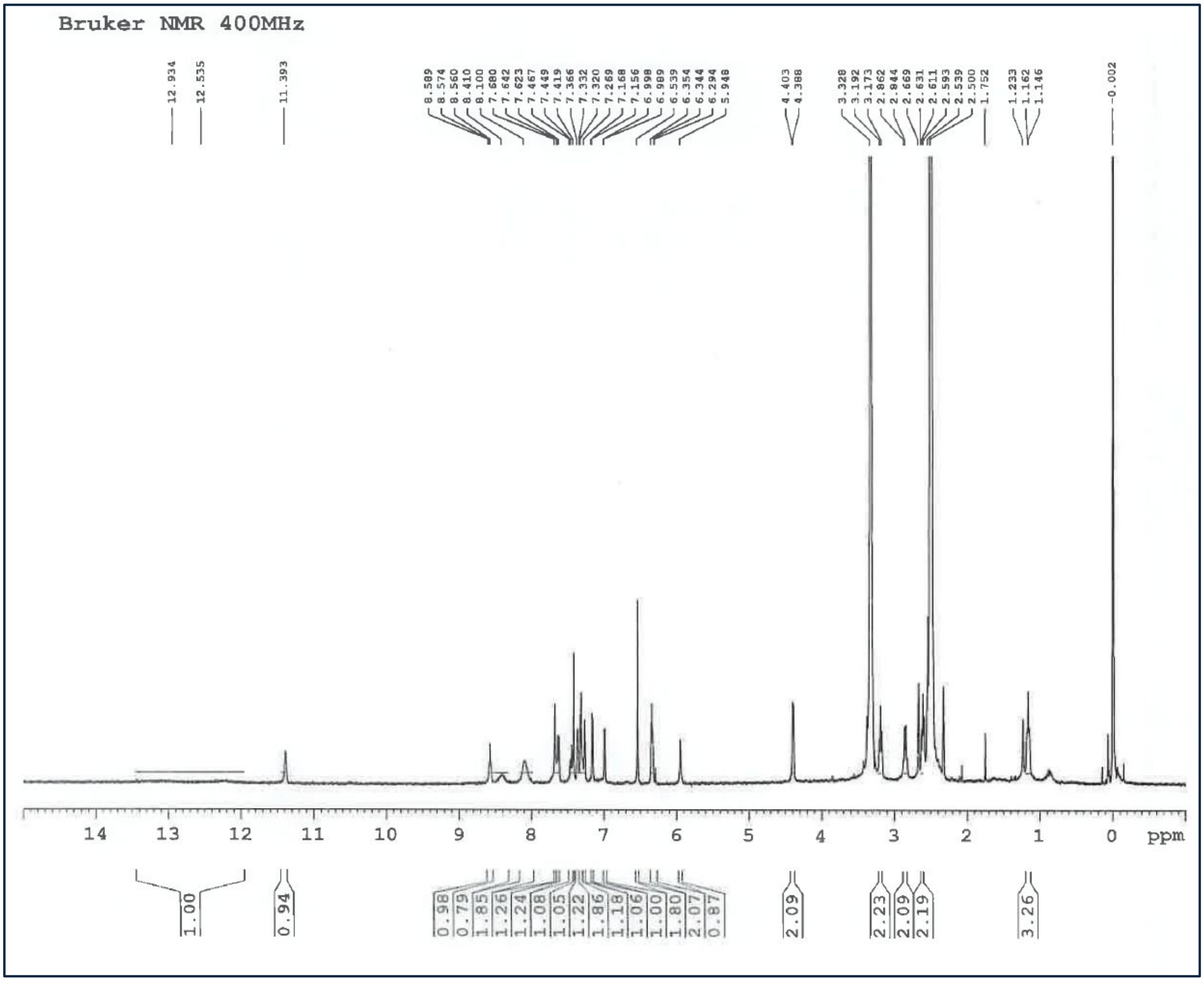

<u>Figure S4</u>. *^{1}H NMR (400 MHz) of BCT2102.*

Figure S5

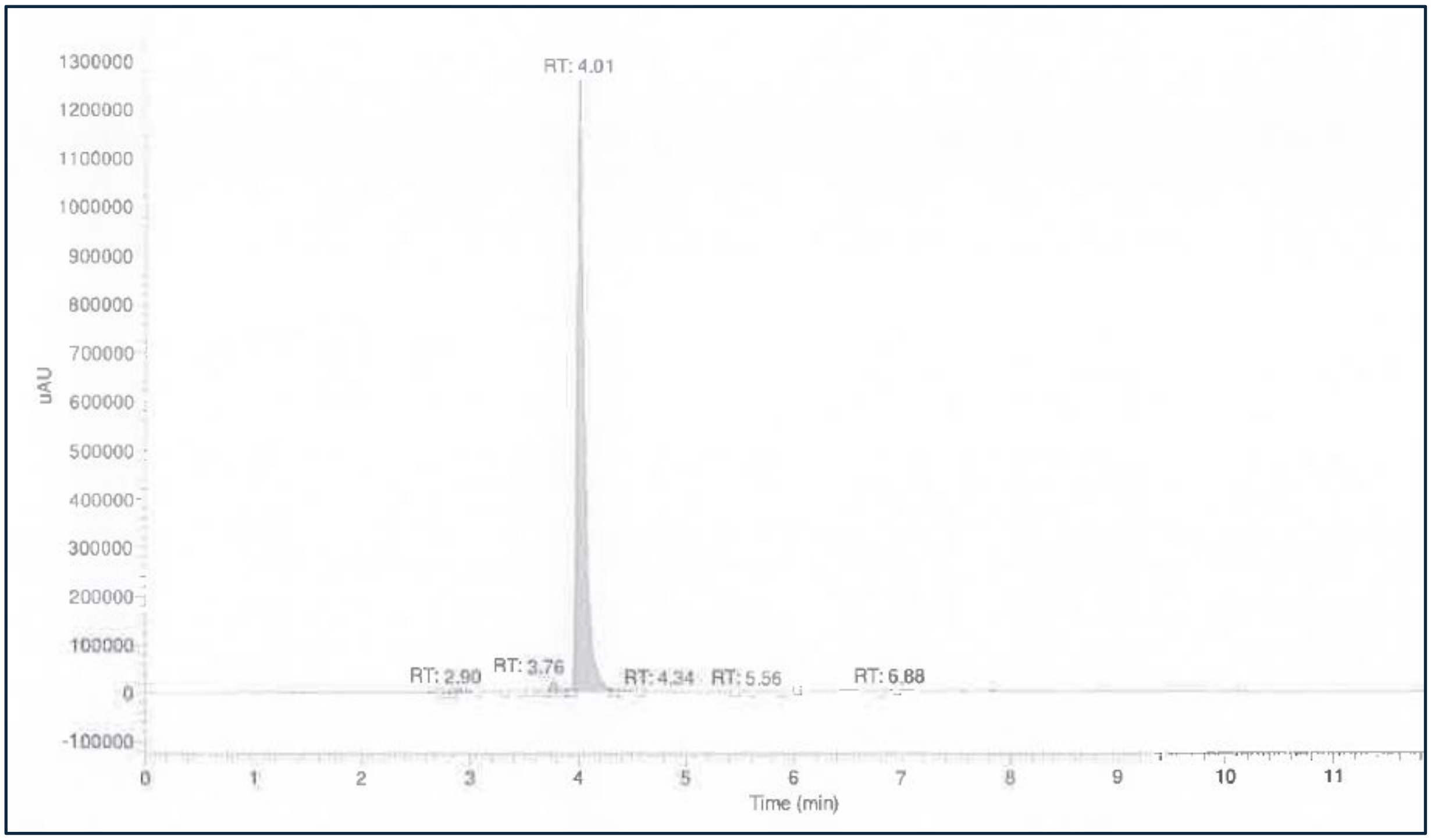

<u>Figure S5</u>. *HPLC chromatogram of BCT2102***.** Purity at peak retention time (4.01 minutes) is 95.39%.

Figure S6

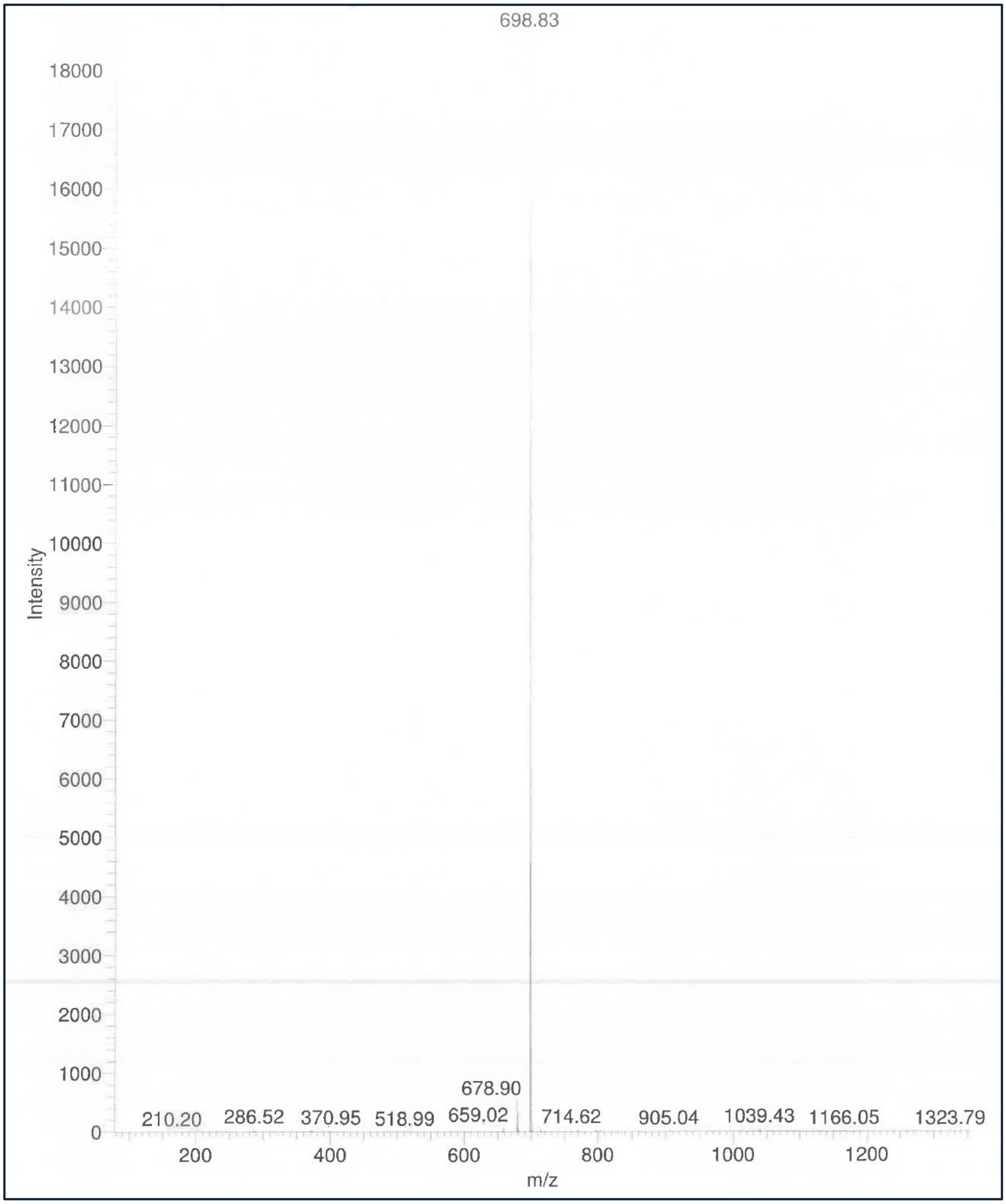

<u>Figure S6</u>. *LC/MS/MS spectra of BCT2102***.** The predominant 698.83 $[M + H]^+$ peak is shown (95.86% purity).

Figure S7

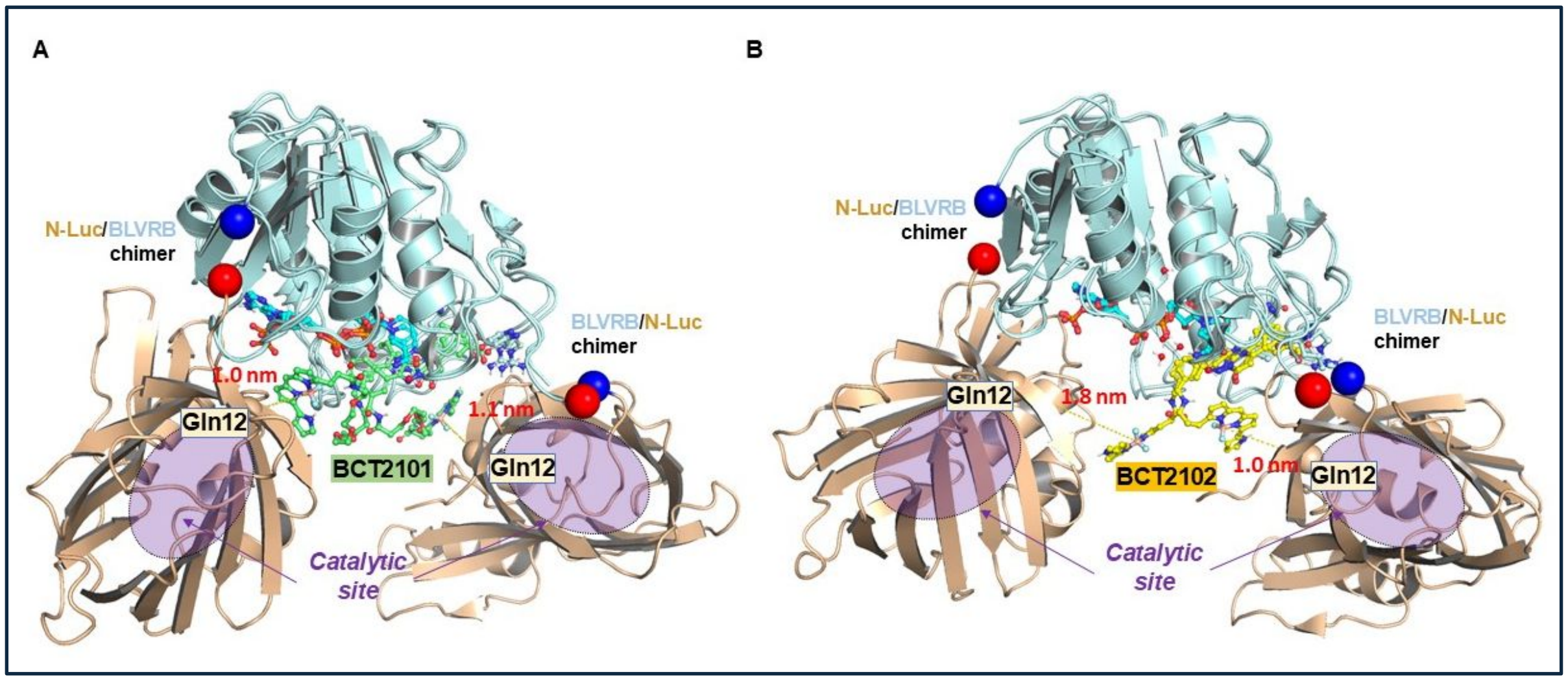

Figure S7. *Computational comparison of BLVRB_NLuc chimers.* **A, B**. Schema demonstrates comparison of the predicted orientations of the NLuc protein (wheat cartoons) with respect to BLVRB (pale blue cartoons) for the BLVRB_NLuc and NLuc_BLVRB chimers. Docked poses of acceptor ligands BCT2101 (*Panel A*) and BCT2102 (*Panel B*) are shown (represented as green and yellow, respectively), along with and $NADP^+$ (blue). The N- and C-terminal residues (Cα carbon) considered to generate the fused chimeric systems are highlighted as blue and red spheres, respectively. Position of the catalytic NLuc residue Gln^{12} is highlighted with a wheat sphere. The nanoluciferase catalytic site is marked with a violet circle. Distances between Gln^{12} (Cα carbon atom) and the fluorophore moiety (B atom) of BCT2101 and BCT2102 are highlighted with black dashed lines.

Figure S8

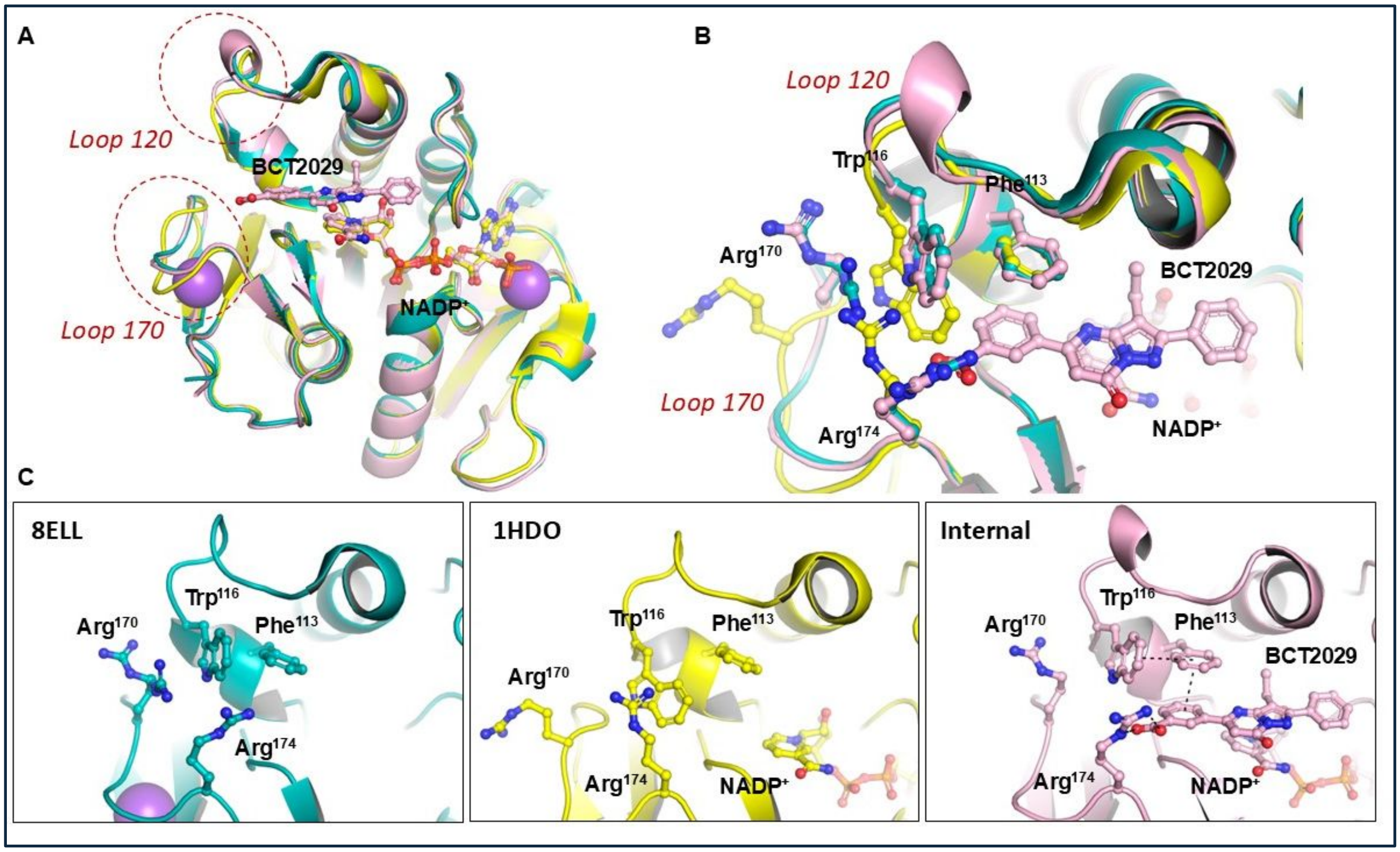

<u>**Figure S8.**</u> *Structural comparison of apo, cofactor-bound, and BLVRB/NADP$^+$/BCT2029 crystal structures.* Superimposition of the crystal structures of *apo* BLVRB (PDB ID 8ELL, resolution 1.52 Å; deep teal cartoons), cofactor-bound BLVRB (PDB ID 1HDO, resolution 1.15 Å; yellow cartoons), and BLVRB/NADP$^+$/BCT2029 (PDB ID 9C16, resolution 1.58 Å; light pink cartoons) are shown in *Panel A*. The cofactor and BCT2029 are shown as stick representations. Loops 120 and 170 are highlighted with dashed circles. *Panel B* close-up view shows the catalytic binding site in the three structures. Key residues defining the binding pocket are displayed as sticks. *Panel C* shows comparison of amino acid side-chain orientations in the *apo*, cofactor-bound, and BLVRB/NADP$^+$/BCT2029 structures. Residues from loops 120 and 170 that directly or indirectly contribute to stabilization of the inhibitor-bound complex are shown as sticks. Protein–ligand and protein–protein interactions are indicated by dashed black lines. Water molecules (where present) were removed for clarity.

Figure S9

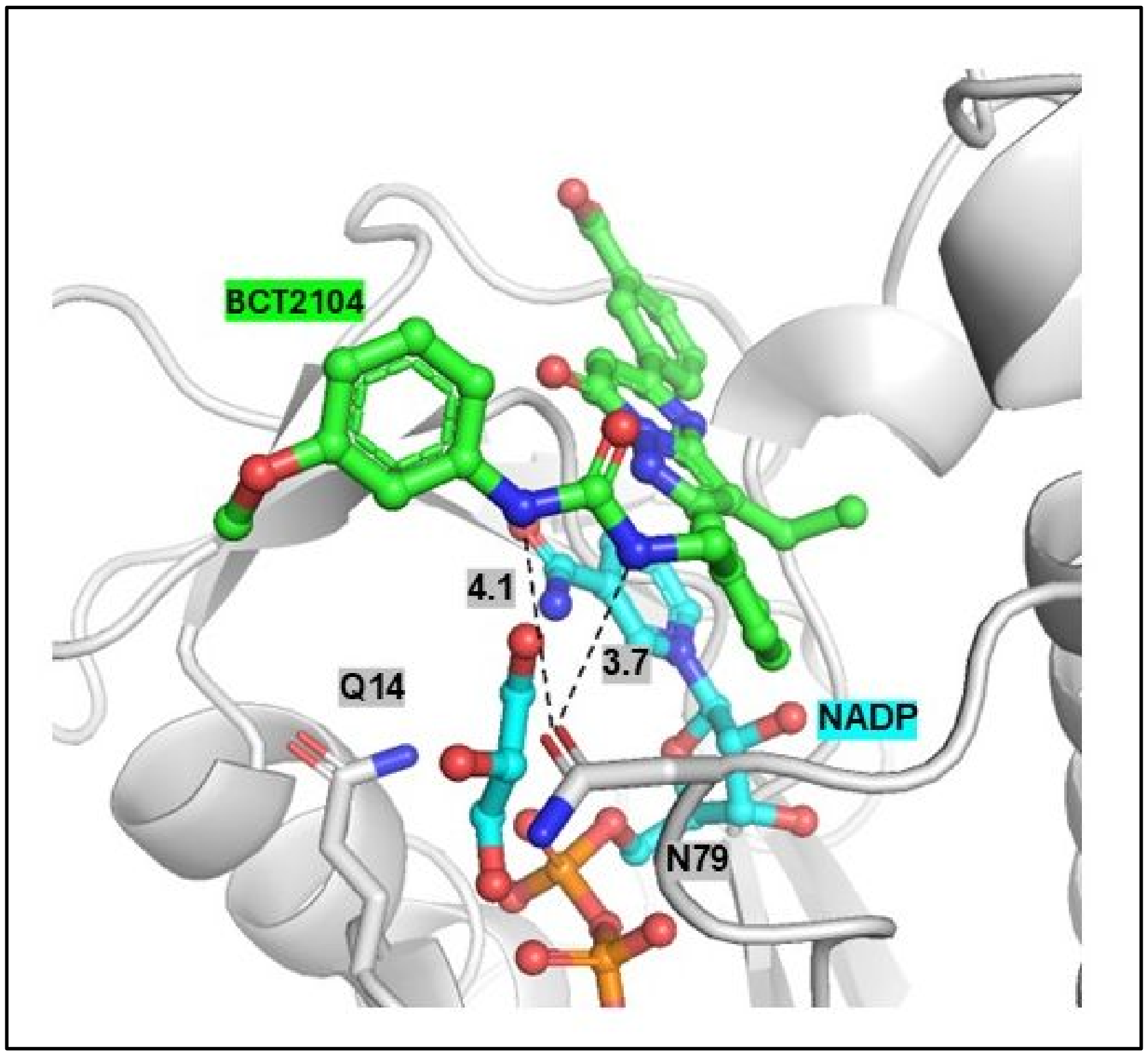

<u>Figure S9</u>. *BCT2104/NADP⁺/BLVRB crystal structure.* The BCT2104/$NADP^+$/BLVRB ternary complex displays a potential hydrogen bonding interaction with Asn79. The disubstituted urea of BCT2104 could engage the Asn79 primary amide which in part explains the increased residence time of BCT2104. Potential hydrogen bonds are labeled with dashed lines and distances are indicated in Angstroms (Å).

Figure S10

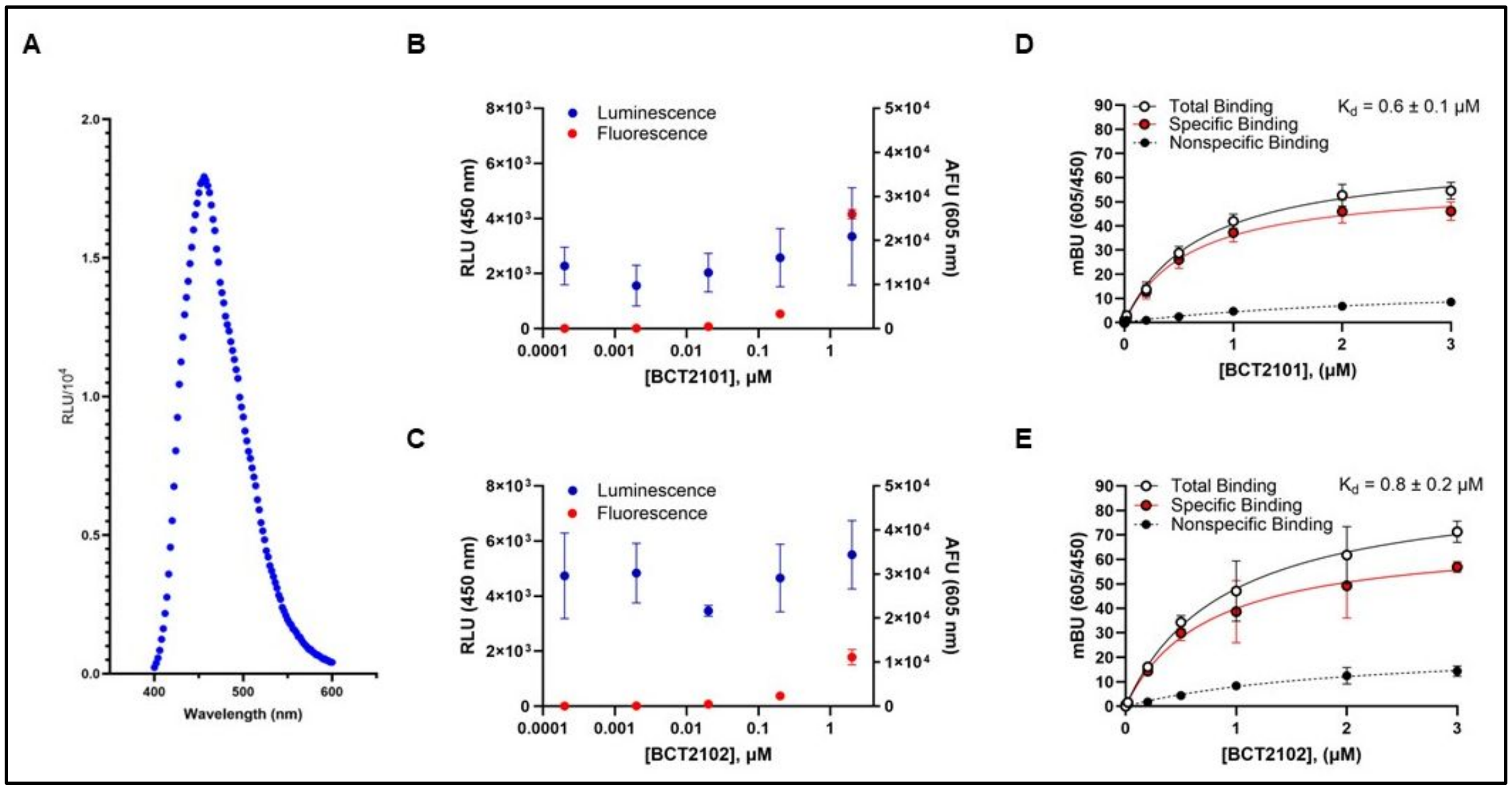

<u>Figure S10.</u> *Characterization of recombinant BRET (rBRET) target engagement assay*. **<u>A</u>**. Luminescence spectra of 50 nM recombinant BLVRB_NLuc (*blue curve*). **<u>B, C.</u>** Raw luminescence (450 nm, *blue circles, left axis*) and fluorescence (605 nm, *red circles, right axis*) signals from BLVRB_NLuc (50 nM) at increasing concentrations of BCT2101 (*Panel B*) or BCT2102 (*Panel C*). Data shown are mean ± SEM (n = 3 independent experiments). **<u>D, E.</u>** Total (*white*), specific (*red*), and nonspecific (black, using 5 μM unlabeled BCT2029) binding of BCT2101 (*Panel D*) and BCT2102 (*Panel E*) using BLVRB_NLuc (50 nM). Apparent tracer dissociation K_ds were determined from saturation binding by fitting specific binding values (total minus nonspecific) to a one-site saturation model using GraphPad Prism (data are the mean ± SEM from N =3 independent experiments).

Figure S11

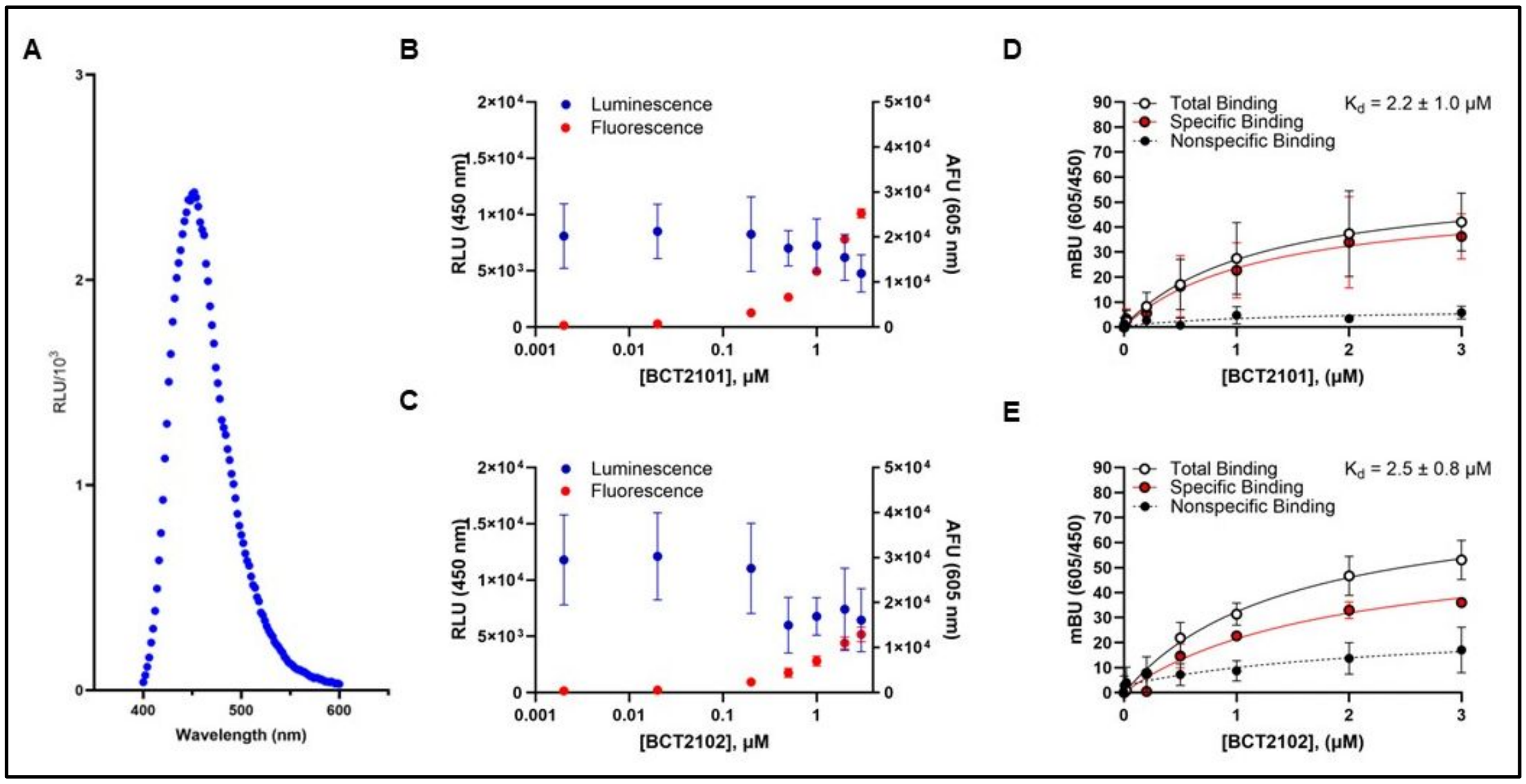

Figure S11. *Cellular nanoBRET characterization in HEK293/BLVRB_NLuc cells.* **A.** Luminescence spectra of HEK293/BLVRB_NLuc cells (2 X10^4, blue curve). **B, C.** Raw luminescence (450 nm (blue circles), *left axis*) and fluorescence (605 nm (red circles), *right* axis) signals from HEK293/BLVRB_NLuc cells at increasing concentrations of BCT2101 (*Panel B*) and BCT2102 (*Panel C*). Data are the mean ± SEM (N = 3 independent experiments). **D, E**. Total (*white circles*), specific (*red circles*), and nonspecific (supplemented with 20 µM BCT2029) binding isotherms of BCT2101 (*Panel D*) and BCT2102 (*Panel E*) are shown. The reported tracer dissociation K_ds were determined from saturation binding by fitting specific binding values (total minus nonspecific) to a one-site saturation model using GraphPad Prism (data are the mean ± SEM from N =3 independent experiments).

Figure S12.

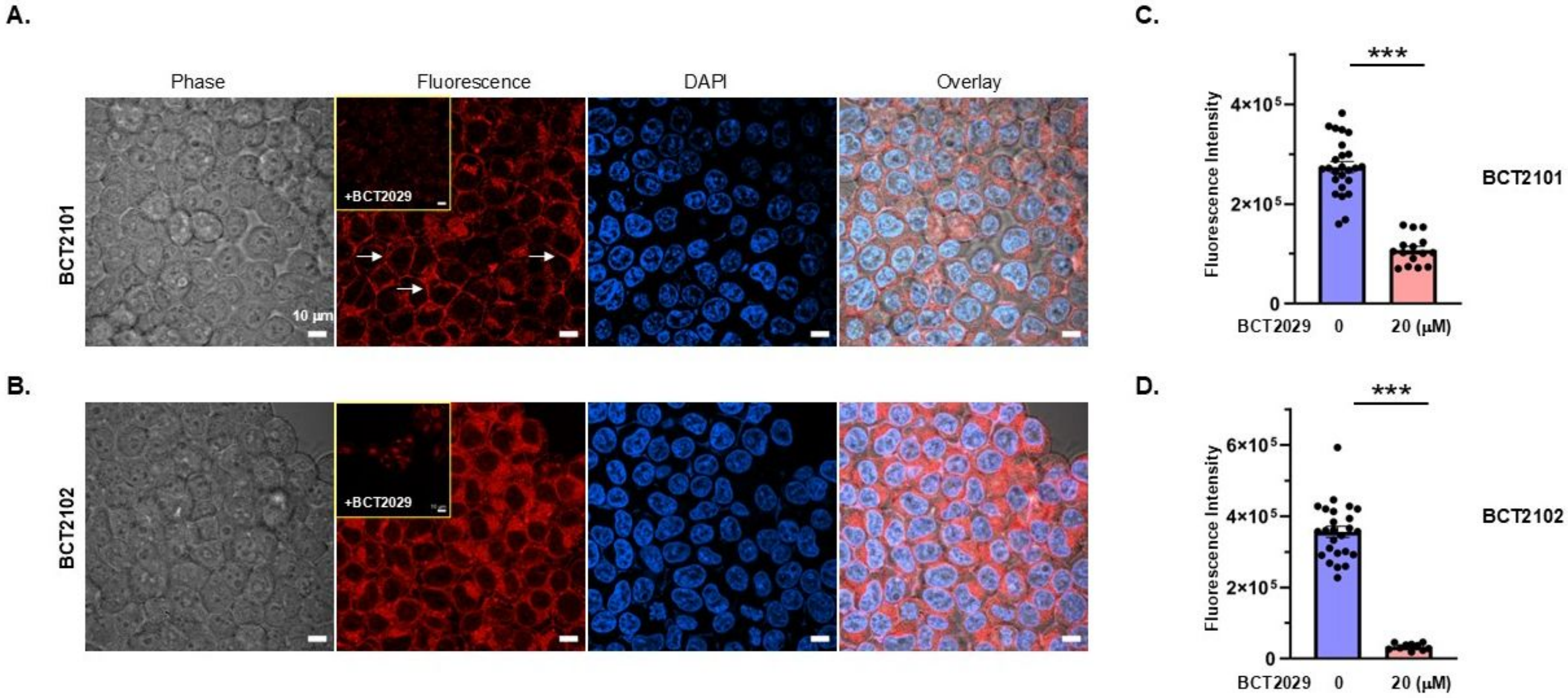

<u>Figure S12</u>. *Affinity ligand live cell imaging of HEK293/BLVRB_NLuc cells.* **<u>A, B.</u>** Adherent cells grown on poly-D-lysine coated glass coverslips were incubated with BCT2101 or BCT2102 (5 μM) for 60 minutes at 37 ^{0}C, washed, and live cell imaging completed by confocal microscopy. Nuclear detection using 10 μM DAPI and phase contrast overlays are shown. The insets in *Panels A* and *B* show fluorescent intensity attenuation in cells simultaneously loaded with BCT2029 (20 μM). Scale bar (10 μm) is shown. **<u>C. D.</u>** Fluorescence intensity was quantified using ImageJ software for cells incubated with BCT2101 (*Panel C*) or BCT2102 (*Panel D*), supplemented without or with unlabeled BCT2029 (20 μM). Bar plots represent fluorescence intensity means ± SEM from 2 independent experiments; p-values ***<0.0001 using unpaired t-test.

Table S1. *X-ray crystallographic data collection and refinement statistics*

**PDB ID**	**13DZ**	**13EA**	**13EB**
Ligand	BCT002104	BCT002066	BCT002109
Wavelength	0.920 Å	0.920 Å	0.920 Å
Resolution range	31.84-1.72 (1.75-1.72)	31.62-1.74 (1.77-1.74)	31.96-1.82 (1.85-1.82)
Space group	*C*2	*C*2	*C*2
Cell constants a, b, c, α, β, γ	76.2 Å, 42.4 Å, 66.7 Å 90°, 107.4°, 90°	75.6 Å, 42.1 Å, 66.3 Å 90°, 107.5°, 90°	75.8 Å, 42.2 Å, 66.8 Å 90°, 106.9°, 90°
Rmerge (all I+ & I-)	0.130 (1.492)	0.118 (1.205)	0.185 (1.642)
Rmeas (all I+ & I-)	0.152 (1.774)	0.137 (1.410)	0.215 (1.900)
Rpim (all I+ & I-)	0.078 (0.948)	0.068 (0.721)	0.108 (0.947)
Total number of observations	81289 (3725)	78898 (3574)	70167 (3492)
Total number unique	21585 (1091)	20461 (988)	18099 (890)
Mean(I)/sd(I)	6.1 (0.9)	7.1 (1.1)	5.3 (0.9)
Completeness (spherical)	99.8 (100.0)	98.8 (97.6)	98.2 (97.7)
Multiplicity	3.8 (3.4)	3.9 (3.6)	3.9 (3.9)
CC(1/2)	0.995 (0.343)	0.996 (0.465)	0.991 (0.397)
**Refinement**			
Resolution range	31.84-1.72 (1.80-1.72)	31.62-1.74 (1.83-1.74)	31.96-1.82 (1.93-1.82)
Reflections used in refinement	21462 (2444)	20437 (2731)	18016 (2743)
Reflections used for R-free	1123 (130)	1047 (142)	907 (151)
R-work	0.195 (0.356)	0.173 (0.288)	0.208 (0.324)
R-free	0.221 (0.402)	0.207 (0.315)	0.245 (0.374)
No. of non-hydrogen atoms	1796	1813	1746
No. of macromolecule atoms	1551	1566	1553
No. of ligand atoms	94	81	91
No. of solvent atoms	151	166	102
No. of Protein residues	211	217	211
RMS(bonds)	0.006	0.007	0.007
RMS(angles)	1.083	0.995	1.075
Ramachandran favored (%)	98.0	98.5	98.5
Ramachandran allowed (%)	1.5	1.0	1.0
Ramachandran outliers (%)	0.5	0.5	0.5
Rotamer outliers (%)	1.19	1.17	0.59
Clashscore	0.93	0.92	2.16
Average B-factor	26.1	23.8	23.5
macromolecules	24.9	22.7	22.8
ligands	29.0	20.7	24.6
solvent	36.6	35.7	33.2

* High resolution shells are shown in parentheses

Table S2. *BLVRB/nanoluciferase chimer sequences**

gga tcc acc atg gcc gtc aag aag atc gcg atc ttc ggc gcc act ggc cag acc ggg ctc
G S T M A V K K I A I F G A T G Q T G L
acc acc ctg gcg cag gcg gtg caa gca ggt tac gaa gtg aca gtg ctg gtg cgg gac tcc
T T L A Q A V Q A G Y E V T V L V R D S
tcc agg ctg cca tca gag ggg ccc cgg ccg gcc cac gtg gta gtg gga gat gtt ctg cag
S R L P S E G P R P A H V V V G D V L Q
gca gcc gat gtg gac aag acc gtg gct ggg cag gac gct gtc atc gtg ctg ctg ggc acc
A A D V D K T V A G Q D A V I V L L G T
cgc aat gac ctc agt ccc acg aca gtg atg tcc gag ggc gcc cgg aac att gtg gca gcc
R N D L S P T T V M S E G A R N I V A A
atg aag gct cat ggt gtg gac aag gtc gtg gcc tgc acc tcg gct ttc ctg ctc tgg gac
M K A H G V D K V V A C T S A F L L W D
cct acc aag gtg ccc cca cga ctg cag gct gtg act gat gac cac atc cgg atg cac aag
P T K V P P R L Q A V T D D H I R M H K
gtg ctg cgg gaa tca ggc ctg aag tac gtg gct gtg atg ccg cca cac ata gga gac cag
V L R E S G L K Y V A V M P P H I G D Q
cca cta act ggg gcg tac aca gtg acc ctg gat gga cga ggg ccc tca agg gtc atc tcc
P L T G A Y T V T L D G R G P S R V I S
aaa cat gac ctg ggc cat ttc atg ctg cgc tgc ctc acc acc gat gag tac gac gga cac
K H D L G H F M L R C L T T D E Y D G H
agc acc tac ccc tcc cac cag tac cag aac atg gtc ttc aca ctc gaa gat ttc gtt ggg
S T Y P S H Q Y Q N M V F T L E D F V G
gac tgg cga cag aca gcc ggc tac aac ctg gac caa gtc ctt gaa cag gga ggt gtg tcc
D W R Q T A G Y N L D Q V L E Q G G V S
agt ttg ttt cag aat ctc ggg gtg tcc gta act ccg atc caa agg att gtc ctg agc ggt
S L F Q N L G V S V T P I Q R I V L S G
gaa aat ggg ctg aag atc gac atc cat gtc atc atc ccg tat gaa ggt ctg agc ggc gac
E N G L K I D I H V I I P Y E G L S G D
caa atg ggc cag atc gaa aaa att ttt aag gtg gtg tac cct gtg gat gat cat cac ttt
Q M G Q I E K I F K V V Y P V D D H H F
aag gtg atc ctg cac tat ggc aca ctg gta atc gac ggg gtt acg ccg aac atg atc gac
K V I L H Y G T L V I D G V T P N M I D
tat ttc gga cgg ccg tat gaa ggc atc gcc gtg ttc gac ggc aaa aag atc act gta aca
Y F G R P Y E G I A V F D G K K I T V T
ggg acc ctg tgg aac ggc aac aaa att atc gac gag cgc ctg atc aac ccc gac ggc tcc
G T L W N G N K I I D E R L I N P D G S
ctg ctg ttc cga gta acc atc aac gga gtg acc ggc tgg cgg ctg tgc gaa cgc att ctg
L L F R V T I N G V T G W R L C E R I L
gcg gaa aac ctg tat ttt cag gga aac agc agc ggc tgg agc cat ccg cag ttt gaa aaa
A **E N L Y F Q G** N S S G W S H P Q F E K
agc agc ggc cat cat cac cat cat cac taa
S S G H H H H H H ***

***Annotations:**

BLVRB

Nanoluciferase

His_6

TEV protease recognition sequence (ENLYFQ/G)

Table S3. *Nanoluciferase/BLVRB chimer sequences**

A. Nanoluciferase/BLVRB chimer*

agc agc cat cat cac cat cat cac agc agc ggc tgg agc cat ccg cag ttt gaa aaa gaa
S S H H H H H H S S G W S H P Q F E K **E**
aac ctg tat ttt cag gga gga tcc aac atg gtc ttc aca ctc gaa gat ttc gtt ggg gac
N L Y F Q G G S N M V F T L E D F V G D
tgg cga cag aca gcc ggc tac aac ctg gac caa gtc ctt gaa cag gga ggt gtg tcc agt
W R Q T A G Y N L D Q V L E Q G G V S S
ttg ttt cag aat ctc ggg gtg tcc gta act ccg atc caa agg att gtc ctg agc ggt gaa
L F Q N L G V S V T P I Q R I V L S G E
aat ggg ctg aag atc gac atc cat gtc atc atc ccg tat gaa ggt ctg agc ggc gac caa
N G L K I D I H V I I P Y E G L S G D Q
atg ggc cag atc gaa aaa att ttt aag gtg gtg tac cct gtg gat gat cat cac ttt aag
M G Q I E K I F K V V Y P V D D H H F K
gtg atc ctg cac tat ggc aca ctg gta atc gac ggg gtt acg ccg aac atg atc gac tat
V I L H Y G T L V I D G V T P N M I D Y
ttc gga cgg ccg tat gaa ggc atc gcc gtg ttc gac ggc aaa aag atc act gta aca ggg
F G R P Y E G I A V F D G K K I T V T G
acc ctg tgg aac ggc aac aaa att atc gac gag cgc ctg atc aac ccc gac ggc tcc ctg
T L W N G N K I I D E R L I N P D G S L
ctg ttc cga gta acc atc aac gga gtg acc ggc tgg cgg ctg tgc gaa cgc att ctg gcg
L F R V T I N G V T G W R L C E R I L A
atg gcc gtc aag aag atc gcg atc ttc ggc gcc act ggc cag acc ggg ctc acc acc ctg
M A V K K I A I F G A T G Q T G L T T L
gcg cag gcg gtg caa gca ggt tac gaa gtg aca gtg ctg gtg cgg gac tcc tcc agg ctg
A Q A V Q A G Y E V T V L V R D S S R L
cca tca gag ggg ccc cgg ccg gcc cac gtg gta gtg gga gat gtt ctg cag gca gcc gat
P S E G P R P A H V V V G D V L Q A A D
gtg gac aag acc gtg gct ggg cag gac gct gtc atc gtg ctg ctg ggc acc cgc aat gac
V D K T V A G Q D A V I V L L G T R N D
ctc agt ccc acg aca gtg atg tcc gag ggc gcc cgg aac att gtg gca gcc atg aag gct
L S P T T V M S E G A R N I V A A M K A
cat ggt gtg gac aag gtc gtg gcc tgc acc tcg gct ttc ctg ctc tgg gac cct acc aag
H G V D K V V A C T S A F L L W D P T K
gtg ccc cca cga ctg cag gct gtg act gat gac cac atc cgg atg cac aag gtg ctg cgg
V P P R L Q A V T D D H I R M H K V L R
gaa tca ggc ctg aag tac gtg gct gtg atg ccg cca cac ata gga gac cag cca cta act
E S G L K Y V A V M P P H I G D Q P L T
ggg gcg tac aca gtg acc ctg gat gga cga ggg ccc tca agg gtc atc tcc aaa cat gac
G A Y T V T L D G R G P S R V I S K H D
ctg ggc cat ttc atg ctg cgc tgc ctc acc acc gat gag tac gac gga cac agc acc tac
L G H F M L R C L T T D E Y D G H S T Y
ccc tcc cac cag tac cag taa
P S H Q Y **Q** ***

***Annotations:**

BLVRB

Nanoluciferase

His_6

TEV protease recognition sequence (ENLYFQ/G)

Table S4. ***Characteristics of BLVRB small molecule inhibitors (N = 11)*** †

			IC$_{50}$			
**Compound**	**Class**	**PDB Structure**	**rBRET [M]$^{-6}$**	**cBRET [M]$^{-6}$**	**DCPIP [M]$^{-6}$**	**Residence time, τ (Min)**
**BCT1028**	Diazabicyclic	9C17¶	0.87 ± 0.10	2.22 ± 0.68	0.13 ± 0.02	64.6 ± 14.7
**BCT1061**	Diazabicyclic		0.11 ± 0.01	0.31 ± 0.04	0.03 ± 0.01	135.9 ± 13.5
**BCT1070**	Diazabicyclic		0.26 ± 0.05	0.95 ± 0.28	0.13 ± 0.01	110.7 ± 17.7
**BCT1072**	Diazabicyclic		0.34 ± 0.08	0.59 ± 0.14	0.10 ± 0.01	115.6 ± 21.0
**BCT2029**	Pyrazolopyrimidinone	9C16¶	0.97 ± 0.17	1.34 ± 0.19	0.09 ± 0.01	57.7 ± 16.6
**BCT2065**	Pyrazolopyrimidinone		26.40 ± 6.40	15.57 ± 0.75	0.08.0 ± 0.02	168.5 ± 20.1
**BCT2066**	Pyrazolopyrimidinone	13EA††	0.12 ± 0.02	0.19 ± 0.11	0.06 ± 0.01	119.1 ± 19.2
**BCT2104**	Pyrazolopyrimidinone	13DZ††	0.85 ± 0.16	0.96 ± 0.02	0.05 ± 0.01	625.0 ± 143.9
**BCT2109**	Pyrazolopyrimidinone	13EB ††	0.50 ± 0.02	2.41 ± 0.02	6.27 ± 0.81	N.D.
**Ataluren**	N/A	7ERB§	1.75 ± 0.48	8.13 ± 0.23	0.38 ± 0.08	48.0 ± 11.6
**Lumichrome**	N/A	1HE5∫	12104.5 ± 10749.5	> 1,000.00	>200,000	N.D.

† Values represent mean ± SEM from N = 3 technical and biological replicates; N. D. – Not done

¶ Reported in Nat Commun 16, 3480 (2025)

†† Reported in current manuscript

§ Reported in J Med Chem 65, 2548 (2022)

∫ Reported in Nat Struct Mol Biol **8**, 215–220 (2001)
